# The Combined Effect of Common Genetic Risk Variants on Circulating Lipoproteins Is Evident in Childhood: A Longitudinal Analysis of the Cardiovascular Risk in Young Finns Study

**DOI:** 10.1371/journal.pone.0146081

**Published:** 2016-01-05

**Authors:** Marie-jeanne Buscot, Costan G. Magnussen, Markus Juonala, Niina Pitkänen, Terho Lehtimäki, Jorma S. A. Viikari, Mika Kähönen, Nina Hutri-Kähönen, Nicholas J. Schork, Olli T. Raitakari, Russell J. Thomson

**Affiliations:** 1 Menzies Institute for Medical Research, University of Tasmania, Hobart, Australia; 2 Research Centre of Applied and Preventive Cardiovascular Medicine, University of Turku, Turku, Finland; 3 Department of Medicine, University of Turku and Division of Medicine, Turku University Hospital, Turku, Finland; 4 Murdoch Children Research Institute, Parkville, Australia; 5 Fimlab Ltd, University of Tampere and Tampere University Hospital, Tampere, Finland; 6 Department of Clinical Physiology, University of Tampere School of Medicine and Tampere University Hospital, Tampere, Finland; 7 Department of Pediatrics, University of Tampere School of Medicine and Tampere University Hospital, Finland; 8 Human Biology, The J. Craig Venter Institute, La Jolla, CA, United States of America; University of Kentucky, UNITED STATES

## Abstract

Low-density lipoprotein cholesterol (LDL-C), high-density lipoprotein cholesterol (HDL-C), and triglycerides (TG) are modifiable risk factors for cardiovascular disease. Several genetic loci for predisposition to abnormal LDL-C, HDL-C and TG have been identified. However, it remains unclear whether these loci are consistently associated with serum lipid levels at each age or with unique developmental trajectories. Therefore, we assessed the association between genome wide association studies (GWAS) derived polygenic genetic risk scores and LDL-C, HDL-C, and triglyceride trajectories from childhood to adulthood using data available from the 27-year European ‘Cardiovascular Risk in Young Finns’ Study. For 2,442 participants, three weighted genetic risk scores (wGRSs) for HDL-C (38 SNPs), LDL-C (14 SNPs) and triglycerides (24 SNPs) were computed and tested for association with serum lipoprotein levels measured up to 8 times between 1980 and 2011. The categorical analyses revealed no clear divergence of blood lipid trajectories over time between wGRSs categories, with participants in the lower wGRS quartiles tending to have average lipoprotein concentrations 30 to 45% lower than those in the upper-quartile wGRS beginning at age 3 years and continuing through to age 49 years (where the upper-quartile wGRS have 4–7 more risk alleles than the lower wGRS group). Continuous analyses, however, revealed a significant but moderate time-dependent genetic interaction for HDL-C levels, with the association between HDL-C and the continuous HDL-C risk score weakening slightly with age. Conversely, in males, the association between the continuous TG genetic risk score and triglycerides levels tended to be lower in childhood and become more pronounced after the age of 25 years. Although the influence of genetic factors on age-specific lipoprotein values and developmental trajectories is complex, our data show that wGRSs are highly predictive of HDL-C, LDL-C, and triglyceride levels at all ages.

## Introduction

Cardiovascular disease (CVD) is the leading cause of death and a major health burden worldwide [1]. Although modified by diet, obesity, lifestyle and other environmental factors, circulating lipoproteins represent a crucial partly heritable risk factor for atherosclerosis and CVD[2, 3]. Notably, elevated levels of total cholesterol and low-density lipoprotein cholesterol (LDL-C), have shown association with preclinical atherosclerosis in children and adolescents [4], significantly contributing to adult atherosclerosis. LDL-C, in particular, plays a major role in the initiation and progression of atherosclerotic lesions [5, 6]. The relevance of high-density lipoprotein cholesterol (HDL-C) and triglycerides (TG) to cardiovascular risk has been extensively debated in the last two decades. Although recent findings have questioned the causal relationship between genetically-defined HDL-C levels and atherosclerosis [7, 8], numerous prospective and case-control epidemiological studies have reported an inverse association between HDL-C levels and the risk of CVD[9–11]. Low HDL-C is therefore considered an independent risk factor for an increased risk of coronary artery disease, although pathways to its potential antiatherogenicity, are still not well understood [12]. High triglyceride (TG) levels are markers for several types of atherogenic lipoproteins involved in atherosclerosis. In contrast to LDL-C, the epidemiologic evidence-base for elevated TG as a CVD risk factor is generally less clear [13–15]. However, recent evidence suggests that genetically-defined plasma TG levels are associated with coronary artery disease risk, even after correcting for confounding effects due to LDL-C or HDL-C levels[16]. In light of these associations, managing dyslipidemia remains a continuing trend both in primary and secondary prevention and risk reduction of CVD worldwide[17].

While the greatest deviations from normal levels of lipoproteins are principally monogenic, the majority of adverse circulating lipid profiles arise as polygenic disorders with a substantial environmental component (e.g. diet, smoking, obesity)[18]. Although dyslipidemia is common, the complex interplay between various genetic and environmental risks that lead to onset and progression of the condition are still poorly understood. In the past 10 years, multiple genetic linkage analyses, candidate gene analyses, and large-scale genome-wide association studies (GWAS) have pinpointed a number of common genetic variants of candidate genes associated with inter-individual variation in plasma lipid levels [2, 19–24], indicating a genetic predisposition to dyslipidemia. Most lipid-associated SNPs (single nucleotide polymorphisms) are characterized by relatively small effect sizes, however some of the reported loci contain genes of clear biological and clinical importance, implicated in established mechanisms of lipoprotein metabolism [25]. Because most individual risk variants only explain a small fraction of those traits’ heritability, the development of multilocus genetic risk scores that combine or accumulate the influence of validated susceptibility markers have proliferated in the hope of improving genetic CVD and other disease risk prediction [26–28].

Unfortunately, it is currently largely unknown whether reported lipid-associated risk alleles have any clinical relevance for a genetic predisposition to elevated adult or childhood lipid levels. With most GWAS leveraging cross-sectional samples from middle-aged adult populations, the relative contribution of these genetic factors to the early stages and development of dyslipidemia between childhood and adulthood remains poorly understood. Moreover, whether identified risk alleles or GWAS-derived genetic risk scores predict lipid trait levels at all ages or whether they are associated with the intra-individual progression of serum lipids over time is unknown.

We examined the combined time-averaged and time-dependent effect of validated genetic risk alleles on lipoprotein trajectories across the life-course in a well-studied prospective population sample: the Cardiovascular Risk in Young Finns Study. Tikkanen et al. have reported population specific cross-sectional associations of 95 GWAS-derived individual SNPs with lipid levels in the Finnish cohort [29]. However, no study to date has investigated the association between lipid genetic risk scores and the development of lipid-trajectories across the life course at the individual level. Our primary aim was to quantify the contribution of a multigenic lipoprotein score to elevated levels of the LDL-C (14 risk SNPs) and triglycerides (24 risk SNPs) as well as depressed levels of the atheroprotective HDL-C (38 risk SNPs) from childhood to adulthood. We also investigated whether a multigenic predisposition to adult dyslipidemia might be modified by a lifestyle trajectory indicator such as the magnitude of BMI change from childhood to adulthood.

## Materials and Methods

### Study sample

The Cardiovascular Risk in Young Finn Study is an ongoing population-based prospective cohort that started in Finland in 1980. It was designed as a collaborative effort between the 5 medical schools in Finland to investigate cardiovascular risk factors and their determinants from childhood to adulthood [30]. A varying number of participants from the original cohort (consisting of 3596 children aged 3 to 18 years in 1980) were measured through middle adulthood (maximum age 49 years) in 2011 for numerous traits related to CVD development, and have as many as seven follow-up measurements (Tables A and B in S1 File, Fig B in S2 File). Among the traits measured at multiple times, serum lipoproteins (plasma concentrations of LDL-C, HDL-C and TG) were collected at baseline and all seven follow-ups. For this study, analyses of the association between polygenic risk scores and circulating lipoprotein trajectories from 1980 to 2011 were pursued. These analyses included up to 2442 participants for whom genetic information was available for each of 76 risk SNPs identified in the literature. Participants reporting use of lipid lowering medication in 2001, 2007 and 2011 were excluded from the analyses (n = 7, 46 and 77 participants excluded respectively for the analysis of LDL-C, HDL-C and TG life course trajectories). Participants or their parents provided written informed consent, and the study was approved by local ethics committees (The Ethics Committee of the Hospital District of Southwest Finland) in agreement with the Declaration of Helsinki.

### Measures

#### Serum lipoproteins

All serum lipid determinations were performed in duplicate on fasting samples using standard methods in the same laboratory. HDL-C was determined enzymatically after precipitation of very low-density lipoprotein and LDL-C with dextran sulfate 500 000 (Olympus System Reagent, Olympus Diagnostica, Hamburg, Germany) in a clinical chemistry analyzer (AU400, Olympus Optical, Mishima, Japan) [31]. The concentration of LDL-C was determined indirectly by the Friedewald formula, so those participants with triglycerides >4.0 mmol/L (n = 32) were not included in the LDL-C evaluation [32]. Specific details on the lipid determination methods used in earlier—[33] and later follow-up studies [34] have been published previously. To adjust for changes in kits and determination methods across study years, lipoprotein levels from early follow-ups were corrected to those measured in the most recent follow-up using correction factor equations, which were determined with linear regression analysis utilizing standardized principal component adjustment [34, 35]. For each lipid, the specific calibrating equations and the dates at which analyzers and reagent suppliers changed along study years are presented in the Appendix 1 of [33] and in [35]. Note: We focused on HDL-C profiles in the present study because measures of other HDL-C fractions were not available at all timepoints in the YF study sample.

Adverse levels of lipoproteins (i.e. abnormally high LDL-C/triglycerides, and low HDL-C), which identify participants at increased risk of developing atherosclerotic CVD (normal risk vs. high risk), were defined using the NCEP adolescent and childhood cut points (for participants under 19 years) [36] and NCEP adult-treatment panel guidelines (for participants 19 years old and older) [37]. The NCEP has not defined desirable and undesirable triglycerides levels for children and adolescents, so high risk triglycerides levels are classified using cutoffs suggested in AAP and AHA pediatric guidelines [38]. The cut points used to define ‘normal’and ‘high risk” lipid levels are shown in S3 Table.

#### Genetic measures and genetic risk scores

In the 2001 follow-up, a, subset of original participants (1,123 males, 1,319 females) were successfully genotyped using a custom Illumina BeadChip containing 670,000 SNPs and CNV probes, for a final list of 546,677 SNPs that passed QC and allele frequency filters. The exact custom content of the probes, as well as initial clustering, filtering, and data exclusion are described by Smith et al. [10]. Genotype imputation was performed using MACH[39] with the HapMap haplotypes as a reference panel (phase II, release 22 CEU, NCBI build 36, dbSNP 126). In the present analyses, we used 38 HDL-C, 24 TG and 14 LDL-C associated SNPs identified by genome wide meta-analysis conducted by the Global Lipids Genetics Consortium (GLGC) on 46 lipid GWAS carried out in over 100,000 European individuals of Caucasian descent [23]. Three composite genetic risk scores (wGRSs) (LDL-C, HDL-C and TG wGRS) were constructed as the arithmetic sums of these 38 HDL-C lowering, 24 TG-raising and14 LDL-C raising alleles respectively, weighted by their effect sizes (in mg.dl^-1^) as established from a published large-scale meta-analysis [23, 28] (S2 Table). To avoid redundancy and overlap of genetic information, in each lipid wGRS, we chose to include only the SNPs with which it showed the strongest independent associations among the 3 lipid traits in the meta-analysis [23]. The variant rs9411489 was not included in the LDL-C wGRS because it was missing on the chip and not available in the HapMap 2 reference panel. For comparability of metrics and to estimate the wGRSs ability to discriminate between extreme lipoprotein phenotypes, participants were categorized into ‘high’ and ‘low’ genetic risk groups categories defined as the cohort-specific lower (25^th^ percentile) and upper (75^th^ percentile) quartile of each composite risk score variables (HDL-C wGRS, TG wGRS and LDL-C wGRS). In each case, the other 50% of participants, lying in the interquartile range, were classified as ‘medium’ genetic risk (Table 1). Histograms of each lipid’s wGRS are presented in Fig A in S2 File. This approach is commonly preferred to case-control dichotomy when investigating the association between genetic factors and disorders implicating quantitative traits continuously distributed over the population (such as dyslipidemia), because it increases the statistical power of testing the variants for association [40].

**Table 1:** Average lipid concentrations in childhood, young adulthood and middle adulthood, across 1980–2011 (in mmol/L), and genetic risk factors considered in the longitudinal lipoprotein profile analyses.

	Males	Females
**HDL Analysis**	(N = 1064**)	(N = 1244**)
Average HDL-C*	1.37 (0.36) (N† = 9043)	1.51 (0.32) (N† = 10540)
**3–15 years**	1.58 (0.34) (N† = 2649)	1.57 (0.30) (N† = 3078)
**18–30 years**	1.28 (0.30) (N† = 3374)	1.52 (0.33) (N† = 3937)
**33–49 years**	1.19 (0.29)(N† = 3020)	1.42 (0.31) (N† = 3525)
**Genetic risk:**		
Average HDL wGRS	32.46 (3.36)	32.62 (3.41)
High score (wGRS >34.84)	N = 253	N = 324
Mid score (30.1<wGRS≤34.8)	N = 541	N = 613
Low score (wGRS ≤30.1)	N = 270	N = 307
**LDL Analysis**	(N = 1121**)	(N = 1314**)
Average LDL-C*	3.22 (0.86) (N† = 9530)	3.17 (0.81) (N† = 10834)
**3–15 years**	3.12 (0.83) (N† = 2781)	3.32 (0.84) (N† = 3255)
**18–30 years**	3.07 (0.85) (N† = 3563)	3.06 (0.80) (N† = 3856)
**33–49 years**	3.41 (0.85) (N† = 3186)	3.07 (0.74) (N† = 3723)
**Genetic risk:**		
Average LDL wGRS	42.1 (6.6*)	41.9 (6.9*)
High score (wGRS >46.1)	N = 278 (25%)	N = 332 (25%)
Mid score (37.5<wGRS ≤46.2)	N = 553 (50%)	N = 665 (50%)
Low score (wGRS ≤37.5)	N = 290 (25%)	N = 317 (25%)
**Triglycerides Analysis**	(N = 1121*)	(N = 1314*)
Average Triglycerides*	1.17 (0.96) (N† = 9513)	1.00 (0.56) (N† = 11148)
**3–15 years**	0.73 (0.32) (N† = 2776)	0.79 (0.34) (N† = 3257)
**18–30 years**	1.17 (0.69) (N† = 3557)	1.06 (0.53) (N† = 4163)
**33–49 years**	1.56 (1.10) (N† = 3180)	1.15 (0.89) (N† = 3728)
**Genetic risk:**		
Average TGwGRS	32.71 (15.81)	131.91 (15.72)
High score (wGRS >142.37)	N = 280 (25%)	N = 334 (25%)
Mid score (121.61<wGRS ≤142.37)	N = 280 (25%)	N = 660(50%)
Low score (wGRS ≤121.61)	N = 275 (25%)	N = 322 (25%)

*Data are sex-specific averages (SD) for lipoprotein concentration and for continuous genetic risks cores (wGRSs) collected for the entire study sample between 1980 and 2011 (the average age of male participants was 24.2 (11.8) years and the average age of female participants was 24.2 (11.8) years over the study period, which was not significantly different). We also present average (SD) lipoprotein levels stratified by age group (i.e. childhood (3–15 years), young adulthood (18–27 years), and middle adulthood (30–49 years). The grouping of wGRSs into categories was based on whole-cohort 25^th^ and 75^th^ percentiles (See methods)

*Abbreviations*: HDL-C, high-density lipoprotein cholesterol; LDL-C, low-density lipoprotein cholesterol.

**Indicates the number of participants included in the longitudinal lipoprotein profile analyses.

^†^ Indicates the number of available measurements for the calculation of each average lipoprotein concentrations.

### Statistical analyses

#### Association between longitudinal lipoprotein profiles and composite genetic risk scores

The principal outcome was the association between the categorical polygenic risk score status (High vs. Low wGRSs) and longitudinal trends in HDL-C, LDL-C and triglycerides levels from 1980 through 2011. To determine whether sex and genetic risk group membership modifies average lipoprotein level or the growth parameters of the participants’ lipoprotein trajectories over time, we used individual growth curve analysis (IGC), an advanced multilevel mixed effect regression technique that allows to model simultaneously inter-individual differences in intra-individual systematic changes over time (i.e. repeated individual measurements) [41–44]. An IGC model comprises 3 main components: (A) the functional form of the response variable, which partitions and quantifies the variance across people and time, (B) the fixed effects (i.e. group-level predictors of change), and (C) the stochastic part of the model, which includes the random effects (i.e. individual effects on growth parameters), and the residual error covariance structure. While there is, in the literature, a few variants in the specification and the procedure of IGC model building (sometimes also referred to as ‘growth curve analysis’ (GCA)[43]), we followed the modeling strategy suggested by Singer and Willett [41], with a few adaptations (see S1 Appendix for a step by step protocol of our modeling approach, and explicit parametrization of the IGC submodels).

Prior IGC analysis, individual empirical growth plots and generalized additive mixed models (GAMMs) were used to explore the functional form (shape) lipid profiles across the life course in the YF cohort [45] and inform the modeling procedure. For each lipid, the IGC analyses then consisted in testing several submodels as follow: (1) an unconditional mean (UM) model (i.e. null model), examining any difference in average lipid levels between individuals, (2) a linear unconditional growth (UG) model (with no group-level predictors), used as a reference to explore the functional shape of the lipid growth overtime (3) two or more higher-order polynomial UG models to test if the lipid rate of change was accelerated or decelerated as subjects aged (i.e. curvilinear age-related change), (4) models for slope(s) variability, to test for random trajectory parameters between participants, (5) a set of models to assess the within-subject error structure of the best UG model to test if incorporating (a) an autoregressive structure with serial correlation, and (b) heterogeneity of the residual error will improve model fit, and finally (5) a conditional growth (CG) model, where wGRSs, sex and their cross-product are introduced as subject-level predictors of each growth parameter variability (i.e. intercept, linear -, quadratic- and cubic (and quartic) growth). CG models allows assessing average wGRSs effect effect at baseline and whether or not there was an age-dependent effect of wGRS score on each lipid’s trajectory parameters (i.e wGRS*age, wGRS*(age)^2^,…). CG models also examine whether individual variability in lipid intercept and slopes estimates can be accounted for by the interaction of wGRSs and sex. Throughout the IGC analyses, when comparing increasingly complex submodels, the improvement in model fit is assessed by likelihood ratio test (LR-test) or using Akaike’s and Bayesian Information Criterion (AIC and BIC). The significance of each estimated model growth parameter in the final CG model is assessed with t-statistics (i.e. defined as the ratio of parameter estimate and SE) (S1 Appendix). A flowchart of the IGC modeling approach is outlined in S1 Fig.

Prior to introducing sex and wGRSs as time-independent predictors of individual variability in lipid trajectories, we considered the need to minimize for confounding by (1) birth cohort and (2) period effect. We tested whether “year of birth” and calendar “year” at examination respectively modified the age-related trajectory of lipids across the life course. This was done by adding the variables (1) birth year (“yob”, categorical variable with 6 levels) and (2) year at follow-up (“year”, centered around baseline (1980)), and their interactions with trajectory parameters to each lipid’s sex-specific UG model (S1 Appendix). Birth cohort does not appear to significantly modify the lipid profiles across the life course in this study sample (i.e. later birth cohorts do not show significantly different lipid trajectories compared to earlier birth cohorts). However, as we found significant linear yearly secular trends for each lipid (see Results), we adjusted for “year” in all subsequent steps of the IGC analyses.

HDL-C and LDL-C distributions were reasonably close to normal (S2 Appendix). Because triglyceride levels showed very long tails skewed to the right, we applied the Box-Cox procedure to determine the optimal transformation to remediate deviations from the assumptions of the linear regression model [46]. As the best transformation (λ = -0.2) was close to the logarithmic case, Natural logarithm was used to transform triglycerides levels prior all analyses. Ages of participants at each measurement were treated as continuous covariates, that we centered around youngest age at baseline (3 years old) to avoid collinearity problems with higher-order polynomial age terms and their interactions in the multilevel mixed models [47]. As an index of fit of the different linear mixed effect models (i.e. final CG models), and to estimate how much the genetic predictors contribute to the variation of the lipoprotein profiles outcome, we used the novel conditional R^2^ and marginal R^2^ for linear mixed models developed by Nakagawa & Schielzeth, 2013 [48] and adapted by Johnson, 2014 [49] to accommodate for random slopes. For a given mixed effect model, the marginal R^2^ describes the proportion of variance explained by the fixed effects alone, while the conditional R^2^, describes the proportion of variance explained by both the fixed and subject- level random factors. In the case of IGC models, which are typically hierarchical mixed models, these two novel coefficients of determination are superior to the pseudo-R^2^ often reported for linear mixed model (i.e. squared correlation between the fitted and observed values) which ignores the variance components at multiple levels of the random factors by choosing to calculate R^2^ relative to only the residual variance. All analyses were performed in R 3.0.1 9 [50] using the nlme 3.1.102 [51] and mgcv [52] packages.

To complement the categorical analyses and make inferences at the population levels, we used a similar age- and sex- adjusted mixed modeling growth curve analysis approach to examine the association of the continuous wGRSs and the longitudinal trends of HDL-C, LDL-C and triglycerides. As per above, main effects (age-averaged) as well as age-dependent effects of continuous lipoprotein risk scores on lipoprotein trajectory parameters were assessed. Z-scores were calculated for the wGRS prior to these continuous analyses, so that for LDL-C, HDL-C and, TG the estimated effects (i.e., the regression parameters: s) indicate the change in mmol/L lipoprotein level per 1-sd change in wGRSs. For triglycerides, the regression coefficient s were exponentiated for ease of interpretation, so that exp(s) correspond to changes in the ratio of the expected triglyceride level per1-sd change in wGRSs.

### Secondary analyses

#### Age- and sex stratified linear regression analysis

To ascertain the age at which the polygenic effect on the lipoproteins is first detectable and examine the strength of association between continuous genetic risk scores wGRSs and lipoprotein levels across age groups, we used sex-specific age-stratified linear regression models adjusted by study year. Trends in the associations between age-groups were assessed using LOESS curves. Additionally, we present a table summarizing sex-specific associations between categorical genetic risk scores and lipid levels at ages 3 years, 15 years, 24 years and 45 or 46 years (S4 Table). The reported mean effect sizes are in mmol/L for the number of risk allele differences between high and low wGRSs for each lipid.

#### Indication of polygenic gene-lifestyle interaction on adult lipoprotein?

Because lifestyle factors relating to weight status (i.e. dietary, exercise, and sedentary habits) are known to strongly correlate with blood lipids, we asked whether the polygenic effect of risk loci on adult lipoproteins might be modified by an individuals’ BMI trajectory from childhood to adulthood. For this, we test for an interaction between continuous wGRS and change in standardized BMI between childhood and adulthood, ΔBMI_i_, in sex- and age-adjusted linear regression models of adult lipoproteins (n = 2100 for adult LDL-C model, n = 2062 for adult triglycerides, and n = 2034 for adult HDL-C model). For each participant _i_, this measure was calculated as:

$$\Delta BMI=z.score\;BMI_{\left(adult\right)i}-average\;\left(z.score\;BMI_{\left(Childhood\right)i}\right)$$ (1)

with z-score BMI_(adult)_ defined as the sex-specific BMI z-scores observed at the latest follow-up attended in adulthood for adults 30 years old or older in 2001, 2007 or 2011 (i.e participants younger than 30 years old in 2001 were excluded); and average(z-score BMI_(childhood)_) defined as the participant’s average of sex-and age-specific BMI z-scores measured at multiple occurrences in childhood (ages at follow-ups ranging 3 to 18). The significance of the interaction term (wGRS *Δ BMI) as a predictor of either adult lipoprotein levels was assessed by a likelihood ratio test.

## Results

Participants’ characteristics for lipoprotein levels and genetic risk scores considered in the longitudinal analyses are shown in Table 1. The difference in the number of risk alleles between subjects in the High wGRS and low wGRS group ranged between 4 and 7 alleles in average alleles for LDL-C and HDL-C, respectively (S4 Table). When stratified by life stages (i.e. childhood (ages 3 to 15), young adulthood (ages 18 to 27) and middle adulthood (ages 30 to 49), average concentrations of each of the three lipoproteins were mostly consistent with pediatric and adult healthy cholesterol and fasting triglycerides levels (NECP guidelines 2010), and the standard deviations were homogeneous over time. For each lipid, a histogram of the continuous wGRSs distribution also showing the quartile stratification into ‘low’, ‘mid’ and ‘high’ genetic risk is shown in Fig A in S2 File. Additional descriptive statistics showed that the wGRSs were not strongly linearly correlated with the lipoproteins overall when ignoring the effect of participants age (Pearson’s r = 0.21 for LDL-C, 0.19 for HDL-C and 0.18 for triglycerides).

### Longitudinal lipoprotein profiles

We found significant decreasing secular trends for LDL-C and TG between 1980 and 2011 (β year_LDL-C_ = -0.09 (se = 0.007) and β year_TG_ = -0.003 (se = 0.0008) respectively, p-values<0.05), but the decreasing trend was not significantly different between males and females. HDL-C, conversely, showed a modest yearly increase (β year_HDL-C_ = 0.005 (se = 0.001), p-value<0.05) for both sexes. However, calendar year at examination did not appear to modify the average age-related trajectories of either lipid in the cohort (i.e. all 3-way year*sex*age-terms interactions were non-significant, p-values >0.05).

**Fig 1:**
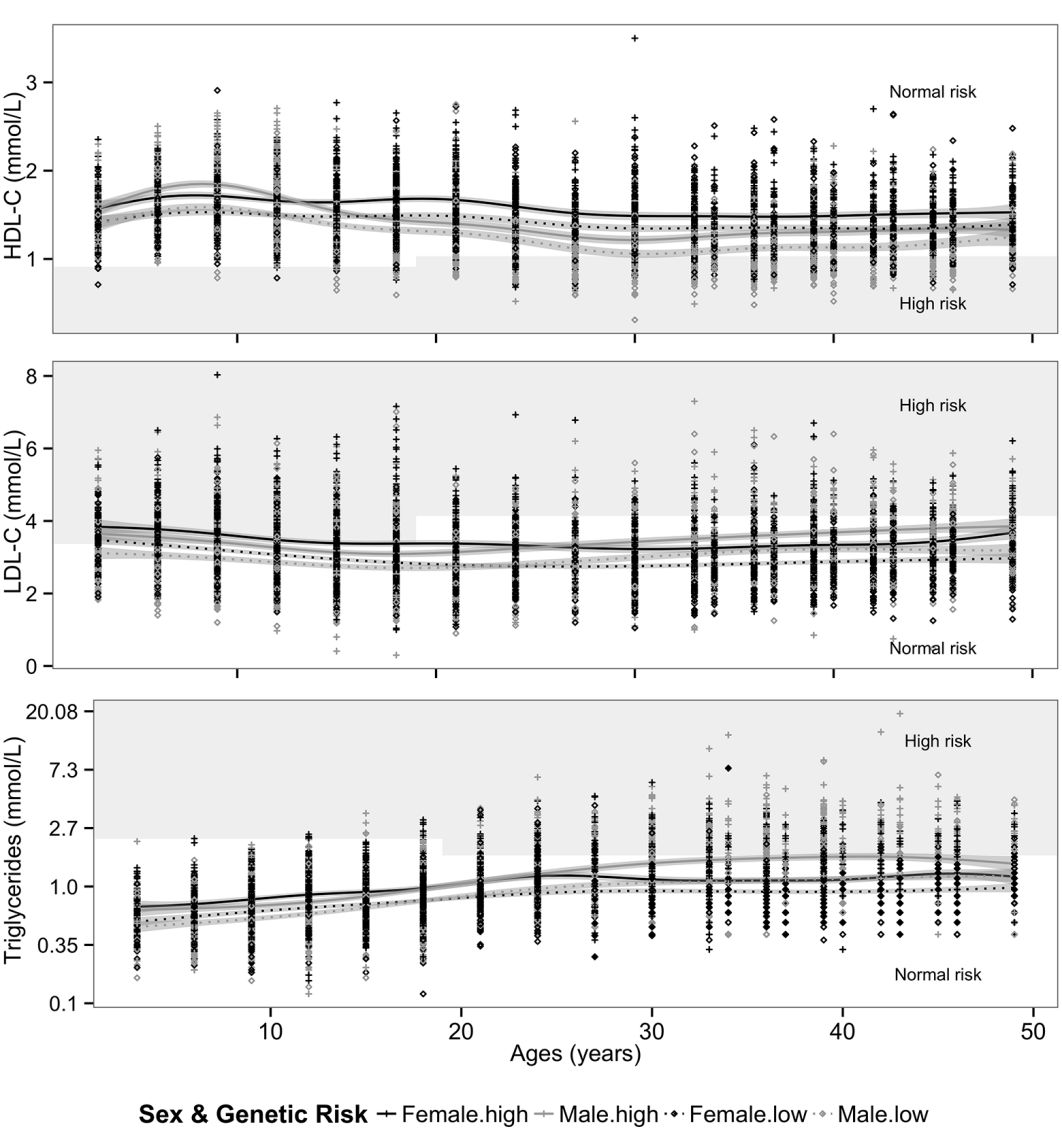
Scatterplot of serum lipoprotein longitudinal profiles of participants according to their sex and wGRSs status (High and Low wGRS*) (N = 2435, N = 2308 and N = 2435 for LDL-C-, HDL-C- and triglyceride profiles respectively). Solid and dotted lines represent estimated sex-specific average age-related lipid trajectories for participants in High and Low genetic risk score, respectively (i.e. prototypical growth curves); grey bands around the growth curves represent approximated 95% prediction CI. Overlaid with the prototypical lipid trajectories are the age-specific cut points for lipoprotein status (normal vs. high risk) as defined by the NCEP adolescent and childhood classification [36] and NCEP adult-treatment panel guidelines [37]). The cut points are represented in grey/white blocks, used to identify those at significantly increased risk of developing atherosclerotic CVD in adulthood. * Mid wGRS risk group are not presented on the figure for the purpose of readability.

The sigmoidal function of age developed by Wineinger et al. 2013 [53] did not fit the lipoprotein profiles in our study sample (lipoprotein _(t)_ ~ intercept + sin(π/2 * ((2 * age_(t)_−max(age)/min(age)) *sex), and the best non-linear fits were achieved by using a 4^th^ polynomial age term for HDL-C profile and 3^rd^ degree polynomial age term LDL-C and triglycerides profiles (i.e. models yielding the lowest AIC and BIC values). Final IGC models assess the effect of sex, wGRSs and their interaction as predictors of the individual variability in lipid growth parameters over the life course. Model selection for the optimal random effects structure revealed that a continuous first order autoregressive correlation structure was needed in each model for the error term, implying that the within-subject correlation between lipid measures drops exponentially with increasing temporal separation. Fig 1 shows the predicted average sex-specific lipid trajectories for the participants in the upper and lower wGRSs quartiles with corresponding 95% confidence intervals, determined from the estimated parameters and SEs of the final IGC models (i.e. prototypical growth curves). Sex-specific prototypical growth curves show that trends in lipoproteins from age 3 to 49 were different for males and females in the cohort. When stratified by sex, participants from ‘high’ or ‘low’ genetic risk groups (upper and lower wGRSs quartiles) showed differences in average levels of each lipoprotein from age 3, but displayed average profiles of globally similar shapes over time (Fig 1).

Participants 10 years and younger already had ‘high-risk’ or close to ‘high-risk ‘average levels of LDL-C (especially for participants in the high genetic risk group, and females in the lowest LDL-C wGRS quartile) as defined by the NCEP pediatric and adolescent cut points [36] (Fig 1). The decrease in HDL-C levels in the cohort was noticeable above the age of 30 years and was more pronounced among males, who in early life had higher average HDL-C levels compared with females, independent of their wGRS status. Similarly, triglyceride profiles in the cohort show a sex-dependent divergence over time, with males tending to exhibit higher average triglyceride levels compared to females from their early to mid 20’s both in the high and low wGRS groups. Fig 1 also suggests the divergence by sex in adulthood is more pronounced in the ‘high risk’ TG wGRS participants, suggesting that males in this group have exacerbated average triglyceride levels from age 25 onwards.

The output of the wGRS group- and sex-adjusted individual growth curve models corroborate the observations that the genetic effect is already present in childhood for each of the three lipids, with significant time independent effects of polygenic genetic risk score categories on baseline lipoprotein levels (Table 2, βs _high-low_ and βs _high-mid_). Indeed, participants in the low and mid LDL-C wGRS group have LDL-C concentrations lowered by 46%, and 23% respectively (p-values <0.0001) as compared to participants in the high LDL-C wGRS. The analysis of HDL-C profiles revealed that participants in the low and mid HDL-C wGRS had average baseline HDL-C levels increased by ~ 16.8% and ~8% respectively as compared to participants in the high risk score group (p-values <0.0005) (Table 2). Similarly, participants in the low and mid TG wGRS had average baseline triglyceride levels lowered by ~ 20% (i.e. log (0.8)) and ~10% (i.e. log (0.9)) respectively as compared to participants in the high risk score group (p-values <0.0005).

**Table 2:** Time-averaged and time-dependent effects of the categorical combined genetic risk scores (HDL-C–, TG–and LDL-C wGRSs) on lipoprotein concentrations (mmol/L) from childhood through adulthood Regression coefficients βs are in % change against the reference group (i.e. High genetic risk group).

	Main wGRS effects^a^		Time-dependent wGRS effects^b^		
Lipoprotein	β(se)	p-val	β(se)	p-val	Goodness of fit
**HDL-C**†					
*High vs*. *Low*	-0.17 (0.01)	0.0001*	L: 0.022 (0.0015)	0.005*	Marginal R^2^: 0.21
			Q: -2.6x10^-4^ (1.1x10^-3^)	0.02*	Conditional R^2^:0.72
			C: 2.5x10^-6^ (3.4x10-^6^)	0.47	
			4^th^: 4.0x10-^7^ (2.4^-^x10^-7^)	0.09	
*High vs*.*Mid*	-0.08 (0.012)	1.0x10^-4^*	L: 0.0014 (0.0009)	0.11	
			Q:- 1.0x10-^4^ (9.0x10^-5^)	0.28	
			C: 2.5x10^-6^ (5.1x10^-6^)	0.40	
			4^th^: 1.0x10-^7^ (2.1x10^-7^)	0.67	
**LDL-C**††					
*High vs*. *Low*	-0.46 (0.04)	1.0x10^-4^*	L:—7.0x10^-3^ (0.002)	0.72	Marginal R^2^: 0.11
			Q: 2.8x10^-5^ (9.5x10^-7^)	0.76	Conditional R^2^: 0.71
			C: 3.0x10^-6^ (6.6x10^-7^)	0.71	
*High vs*.*Mid*	-0.23 (0.03)	1.0x10^-4^*	L: 6.1x10^-2^ (1.9x10^-2^)	0.52	
			Q: 6.3x10^-5^ (6.1x10^-6^)	0.45	
			C: 1.3x10^-9^ (1.0x10^-9^)	0.94	
**Triglycerides**†††					
*High vs*. *Low*	0.80 (0.025)	0.0001*	L: 0.995 (0.001)	0.11	Marginal R^2^: 0.18
			Q: 0.99 (6.8x10^-5^)	0.08	Conditional R^2^:0.68
			C: 1.00 (4.9x10^-7^)	0.37	
*High vs*.*Mid*	0.90 (0.022)	1.0x10^-4^*	L: 0.999 (0.001)	0.15	
			Q:- 0.997 (5.8x10^-5^)	0.09	

*Abbreviations*: HDL-C, high-density lipoprotein cholesterol; LDL-C, low-density lipoprotein cholesterol; *High/Mid/ Low*, categorical genetic risk score groups (for either lipoprotein trait); *L*, *Q*, *C and 4*^*th*^, Linear/quadratic/cubic/ and quartic rate of change (in either lipoprotein concentration as a function of age).

^†^ / ^††^ / ^†††^ wGRS effects refer to the combined effect of the 38-, 14- and 24 SNPs associated respectively to HDL-C, LDL-C and fasting triglycerides levels (see methods).

* Indicates that the estimated regression parameter is significant at the 0.05 significance level.

^a,b^ For ease of interpretation of the estimates of main and time-dependent effects of wGRSs, all age terms were centered around youngest childhood age at baseline (1980) in the cohort (3 years old) prior regression analysis. For triglycerides, regressions coefficients βs of main and time-dependent effects were exponentiated so they are presented in the original scale for ease of interpretation.

Analyses of interactions terms revealed no time-dependent polygenic effects of the 14 risk SNPs on circulating LDL-C, implying that there is no worsening effect of LDL-C levels over time among those belonging to the high genetic risk group compared with those in the low or mid genetic risk group (all linear, quadratic and cubic β_[wGRS *age_ interactions *p*-values >0.3). On average at baseline, males had 7% higher LDL-C levels and 15% lower HDL-C levels compared with females (p-values <0.004). Sex appears to be the variable that drives the longitudinal trajectory of LDL-C levels in this cohort (β_sexMale*f(age)_ = 0.019, se = 0.0007, *p*-values <0.001), rather than wGRS group membership (linear, quadratic, and cubic rate of change not significant, Table 2).

For HDL-C however, we found a significant linear and quadratic age-dependent interactions between participants belonging to the high and low wGRS group (β_[wGRS *f(age)]_ = 0.022 and β_[wGRS *f(age-square)_ = -2.6 x10^-04^) (Table 2), suggesting that genetic group membership is a modifier of the HDL-C trajectory. The positive effect of high wGRS on the linear age term implies that, for children in the high wGRS category, the effect of the combined variants leads to an initial increase slightly faster (by 2.2%) per year as compared to children in the low wGRS group (i.e. slightly steeper linear increase). Similarly, the negative effect on the quadratic rate of change also indicates that for participants in the high wGRS group, the positive genetic effect on HDL-C levels will decelerate in time slightly slower (by 0.026%) than it does in the lowGRS group at around adolescence. This small difference in HDL-C trajectories between high and low wGRS groups is not easily distinguished in Fig 1, as the modifying effect of the variants on the trajectory parameters is relatively mild. These age-dependent interactions were not modified by sex (all 3-way interactions were not significant p>0.05), so that the effect of wGRS categories on HDL-C trajectory parameters were not significantly different in males and females.

For triglycerides, the linear, quadratic, and cubic age-dependent interactions between participants belonging to the high and low wGRS group are not significant in females (Table 2), however, for males, genetic risk group membership modified the linear change if triglycerides (3-way interactions between linear age-dependent change rate, sex, and TG wGRS group membership) were significant both for low vs. high genetic risk group (expβ_[male*wGRS *f(age)]_ = 0.96, se = 0.003, p-value = 0.0009), and for mid vs. high genetic risk group (exp β_[male*wGRS *f(age)]_ = 0.99, se = 0.002, p-value = 0.01). That is, males belonging to the high genetic risk group will tend to have a linear increase in TG levels by 4% (i.e. log(0.96)) and by 1% (i.e. log(0.99) for participants in the mid genetic risk group). As these effects are also moderate, it does not result in strong divergences in the prototypical triglyceride trajectories (Fig 1).

**Fig 2:**
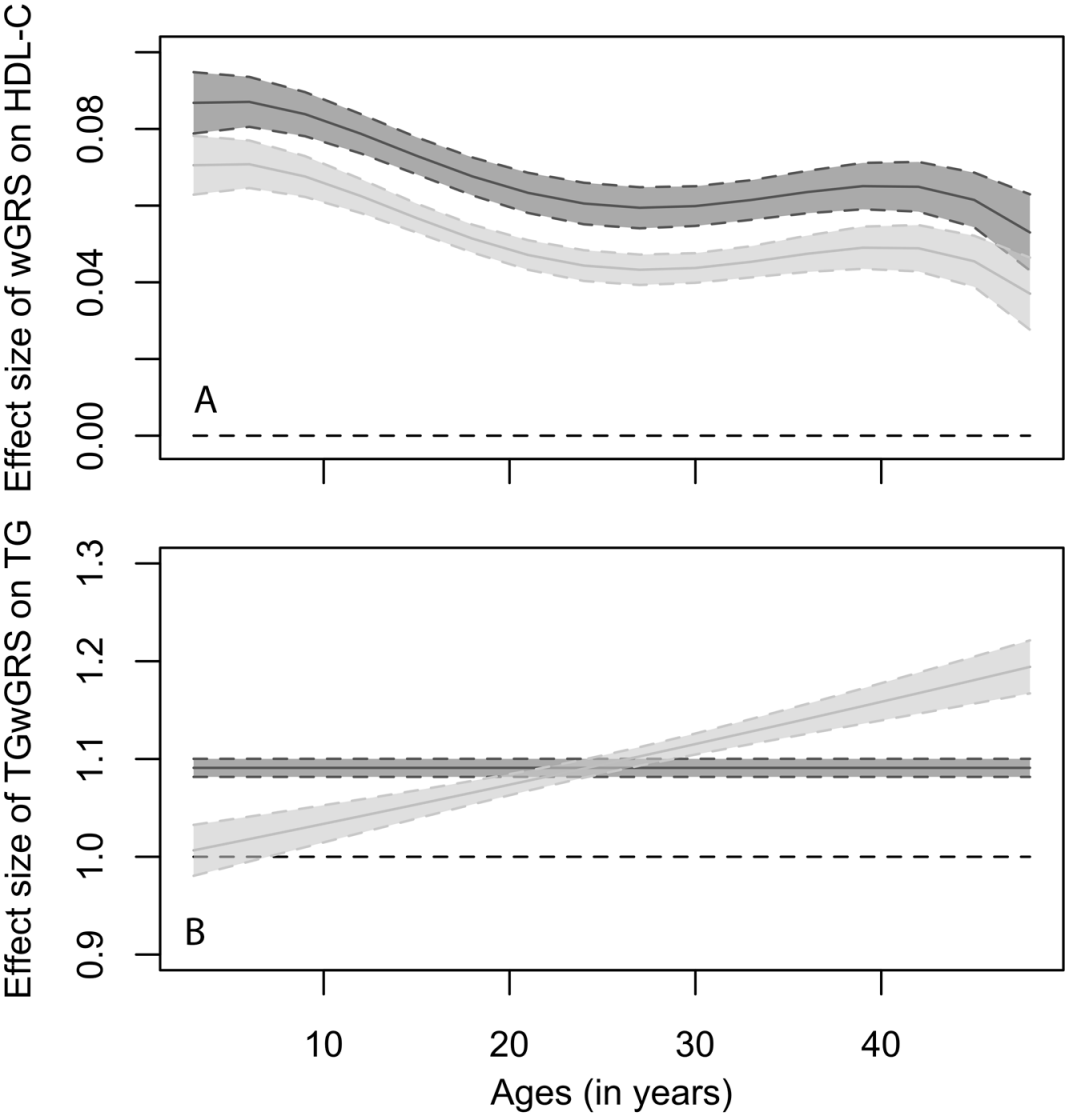
Sex-specific marginal effect* and 95% CI of (A) combined continuous HDL-C wGRS on HDL-C levels (effect size expressed in mmol/L lipoprotein level change per 1-sd change in wGRSs); and (B) combined continuous TG wGRS on fasting triglyceride levels (effect size expressed in odds ratio lipoprotein level change per 1-sd change in wGRSs). Colour code: dark grey; females, light grey; males. (*Plotted marginal effect includes the significant linear slope, quadratic and quartic rates of change (cubic trajectory parameter not significant in the final model); Horizontal black dashed line shows where the slopes are not significantly different from zero).

When using the wGRSs values as continuous predictors, the final IGC models for the age-related trajectories of LDL-C, HDL-C and triglycerides profiles achieved a conditional R^2^ of 0.64, 0.68 and 0.61 respectively, with the fixed predictors (age, sex, and continuous wGRS) jointly accounting for 12%, 27% and 19% of deviance respectively in each model. Consistent with the categorical analyses, the time-averaged genetic effect of the combined genetic risk score on lipoprotein profiles was significant (p<0.0001) for all traits (Table 3). The polygenic effect size was stronger for LDL-C associated risk SNPs, with LDL-C wGRS increasing LDL-C levels by 18.2% per SD increase in score (as compared to a 6.8% increase in HDL-C levels per SD increase in wHDL-C GRS). Additionally, wHDL-C GRS was a significant predictor of the linear, quadratic and quartic changes in HDL-C levels over time (p-values <0.01, Table 3), implying that the slope of the genetic risk score variable on HDL-C concentration changes as participants age from childhood through adulthood. These time-dependent interactions are best visualized by computing and plotting the marginal effect of the combined genetic risk score on HDL-C levels (Fig 2A). The downward trends of the slopes of the continuous risk score on HDL-C level with age for both males and females, suggests that the association between HDL-C levels and of the 38 HDL-C risk SNPs gets weaker as participants aged in this population, although the combined genetic effect of these loci on HDL-C was consistently stronger among females.

**Table 3:** Time-averaged and time-dependent effects of the continuous combined genetic risk scores (HDL-C–, LDL-C—and TG wGRSs) on lipoprotein concentrations (in mmol/L) from childhood through adulthood.

	Main wGRS effects^a^		Time-dependent wGRS effects^b^		
Lipoprotein	β(se)**	p-val	β(se)**	p-val	Goodness of fit
**HDL-C** †	0.0064(0.002)	1.0x10^-4^*	L: -6.1 x10^-3^ (2.0 x10^-4^)	0.003 *	Marginal R^2^: 0.27
			Q: 2.0 x10^-5^ (4.1 x10^-6^)	0.014*	Conditional R^2^: 0.68
			C: 4.0 x10^-6^ (1.2 x10^-7^)	0.07	
			4^th^: -1.0 x10^-7^ (7.8 x10^-9^)	0.05.	
**LDL-C**††	0.182 (0.001)	0.0003*	L: 2.7x10^-4^ (1.2 x10^-3^)	0.85	Marginal R^2:^ 0.13
			Q: -9.5 x10^-5^ (1.5x10^-4^)	0.44	Conditional R^2^: 0.64
			C: 3.0 x10^-6^ (2.6 x10^-6^)	0.23	
**Triglycerides**†††	1.094 (0.008)	1.0x10^-4^*	L: 1.0 (9.8 x10^-4^)	0.47	Marginal R^2^: 0.20
			Q: 1.0(8.0 x10^-5^)	0.31	Conditional R^2^: 0.65
			C: 0.99 (1.7 x10^-7^)	0.20	

*Abbreviations*: HDL-C, high-density lipoprotein cholesterol; LDL-C, low-density lipoprotein cholesterol; *L*, *Q*, *C and 4*^*th*^, Linear/quadratic/cubic/ and quartic rate of change (in either lipoprotein concentration as a function of age).

^†^ / ^††^ / ^†††^ wGRS effects refer to the combined effect of the 38-, 14- and 24 SNPs associated respectively to HDL-C, LDL-C and fasting triglycerides levels (see methods).

* Indicates that the estimated regression parameter is significant at the 0.05 significance level.

^a,b^ For ease of interpretation of the estimates of main and time-dependent effects of wGRSs, all age terms were centered around youngest childhood age at baseline (1980) in the cohort (3 years old) prior regression analysis.

**Regression coefficient βs are in mmol/L per 1-sd change in wGRS for HDL-C and LDL-C. For triglycerides, regressions coefficients βs of main and time-dependent effects were exponentiated so they are presented in the original scale for ease of interpretation.

For triglycerides, continuous analyses revealed that while on average, TG wGRS did not modify the linear, quadratic or cubic change in triglyceride level, but when clustering participant by sex, TG wGRS effect on the linear change rate in triglyceride levels became significant for males (three way interaction exp _βwTGgrs24*sexMale*f(age)_ = 1.0038, se = 0.0011, p-value = 0.001). This difference between sexes can also be visualised by plotting the marginal effect of the TG combined genetic risk score on triglyceride levels (Fig 2B). For females, a 1-sd increase in risk score will result in 9% higher serum triglyceride on average (regardless of their age; log (1.1) = 0.09). For males, the effect size of TG genetic risk score is age-dependent and lower in childhood than for females, it increases linearly to become larger in females from age 25 onwards, reaching 13% at age 49 years (log(1.14) = 0.13).

### Age-stratified linear regression

**Fig 3:**
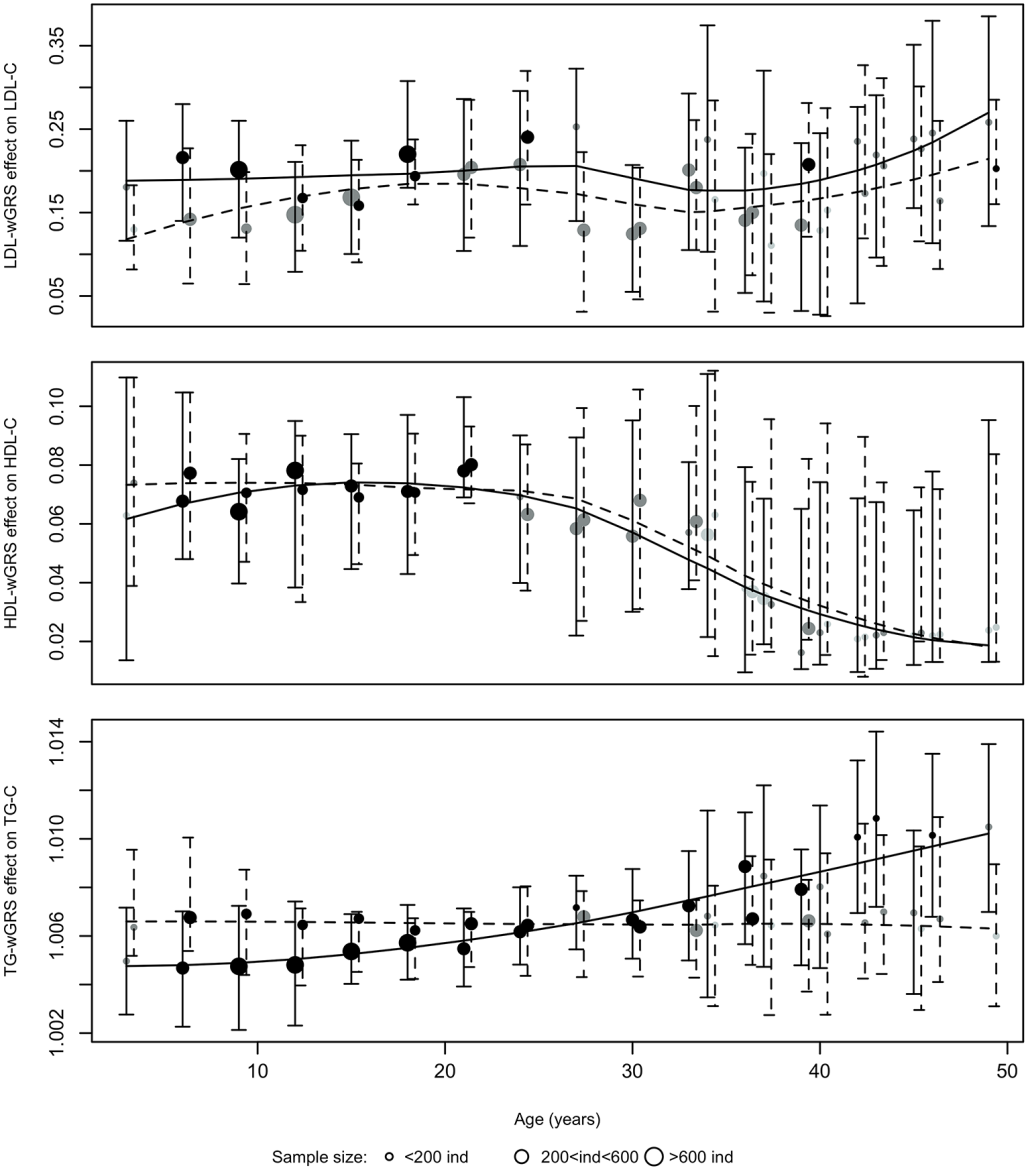
Age- and sex- stratified estimated effects of LDL-C wGRS (upper panel), HDL-C wGRS (middle panel) and TG wGRS (lower panel) on LDL-C, HDL-C and triglycerides levels respectively with color coded significance levels and studentized bootstrapped non-parametric 95% CI. For each age, the continuous error bars correspond to males and the dashed error bars directly next to them correspond to female models. Effect sizes are in mmol/L change per 1-sd change in wGRS for LDL-C and HDL-C and in odds ratio lipoprotein level change per 1-sd change in wGRS for triglycerides. Point sizes of the beta estimates reflect sample size (number of participants included in each age- and sex-specific regression analysis) Parameter estimates significance: Lightgrey, 0.05<p-val<0.01; Darkgrey, 1.x10^-3^<p-val<1.X10^-6^; Black, p-val≤1.X10^-6^. Black lines: smooth trend curves fitted by LOESS (Locally weighted non-parametric regression) to help visualise trends in the cross-sectional associations.

Cross sectional analyses confirmed that combined wGRSs were significantly associated with the three lipoproteins at all ages for both sexes (Fig 3, all p-values <0.05). Confirming that the joint effect of the 14 risk SNPs on LDL-C levels is consistent across time, the fitting of a LOESS regression lines to the ages- and sex-specific regression parameters does not reveal any striking trend over time (Fig 3, upper panel). We suggest that the variations in effect sizes between different age-groups were mostly attributable to differences in sample sizes and differences in the number of birth cohorts used in the regression analysis (only one cohort for the 3 year old age-group, against a mix of up to 5 cohorts for the 15 and 18 year old group). The age- and sex- stratified analysis of the association between HDL-C levels and HDL-C genetic risk however, confirmed that the effect size of HDL-C wGRS on HDL-C decreased almost by half on-average for age-groups >30 both in males and females. For triglycerides, the results of the age-stratified regression analysis are consistent with the results of the individual growth curve analyses, showing very stable effect estimates for females throughout age groups, and a clear upward trend in effect size estimates for males. Sex-specific mean effect sizes for categorical wGRS at chosen ages (i.e. 3, 15 24 and 45/46 years) are consistent with the results of the sex-and stratified cross sectional analyses of the continuous risk scores (S4 Table).

### Evidence for polygenic gene-lifestyle interaction on adult lipoprotein levels

Change in BMI z-score between adulthood and childhood (Δ BMI) was highly predictive of each adult lipoprotein levels independently of the wGRSs in multivariable models adjusted for sex and age at baseline and follow-up (S1 Table). However, the LR tests revealed no evidence for gene-lifestyle interactions (Δ BMI *wGRSs) on the adult lipoprotein concentrations, as the combined effect of candidate genes on adult lipoprotein levels does not appear to be modulated by the trajectory of BMI from childhood in this cohort (χ^2^(df = 1) = 0.24 p = 0.62; χ^2^(df = 1) = 2.04, p-value = 0.15 and χ^2^(df = 1) = 1.17 p-value = 0.11 for HDL-C, LDL-C, and triglycerides respectively).

## Discussion

This is the first study to investigate the combined effect of dyslipidaemia–predisposing variants on longitudinal blood lipoprotein profiles from childhood to adulthood. Rather than considering individual-validated risk lipid-SNPs alone, usually characterized by weak to moderate effect sizes in large lipid GWASs, our approach used weighted genetic risk scores combining multiple loci identified in meta-analyses of large lipid GWAS. Collectively, our findings suggest that genetic factors influence age-specific lipoprotein values and developmental trajectories already from the age of 3 years.

The use of polygenic risk scores has become increasingly popular in recent years for the purpose of genetic prediction of a number of quantitative traits, with the increasing recognition that a substantial part of heritability comes from many susceptibility markers individually characterized by low predictive power [26]. For circulating lipids in particular, when combined, multiple common genetic variants with small effects on circulating lipid levels, were reported to be highly predictive of individual trait measures [28] and showed association with subclinical and clinical cardiovascular outcomes [27]. Despite these promising associations between SNPs (or genetic risk scores) and lipoproteins, susceptibility alleles are often identified from cross-sectional adult GWASs, and it remains unclear whether the within-individual level of genetic risk carried by these variants is stable through life or changes with age. This uncertainty greatly impedes the assessment of the abilities of individual SNPs (or polygenic GWAS-derived risk score) for prediction of quantitative traits collected across the lifecourse and their clinical usefulness in the primary prevention of dyslipidemia.

Independent of participants age–related lipid trends, we found significant decreasing secular linear trends in LDL-C and triglycerides across the 31 follow-up years, consistent with what has been reported in previous studies [54]. These trends may be partially due to a wider use of statins and/ or improvement in diet in the last decades.

Multilevel individual growth curve analysis revealed an expected difference in lipoprotein profiles from childhood to adulthood between males and females, and that while baseline HDL-C, LDL-C and triglycerides levels were not significantly different between sexes (age 3), the actual trajectories of lipid levels from childhood to adulthood were largely sex-driven. We observed a time-averaged association between lipid wGRSs and each of the three serum lipid levels, implying that composite genetic risk scores were robust predictors of average lipoprotein levels, as well as predictors of lipoprotein in childhood (from age 3 at baseline). The time-averaged effect of the 14 risk alleles on LDL-C levels was stronger than the effect of the 38 risk SNPs on HDL-C levels on average, suggesting that the genetic predisposition to high serum LDL-C is stronger compared to a predisposition to low HDL-C levels and high triglycerides levels. The LDL-C wGRSs did not modify the linear, quadratic or cubic trajectory of lipoprotein level, so that the combined effect of the 14 candidate SNPs is not only present in young childhood, but also consistent across a person’s life course as she ages (from age 3 to 49 years). Categorical analyses revealed that the 38 risk SNPs modified the linear and quadratic component of HDL-C trajectory over time, although the effect sizes of the age-dependent terms were small, such that it does not translate to clearly divergent profiles between high- and low-risk participants. However, when plotting the estimated marginal effect of HDL-C wGRS over time (within-individual), for a given male participant the magnitude of the effect of the combined genetic risk score on his HDL-C levels will attenuate with age. This is also confirmed by the cross sectional age-stratified analyses, where childhood HDL-C levels were strongly related to genetics, but where the main effect of HDL-C wGRS diminished by half in age-groups over 30 years old. Although significant at all ages, the collective effect of the HDL-C risk score on HDL-C levels becomes weaker as participants age, with environmental, lifestyle and behavioural factors such as diet, smoking, physical activity, potentially becoming more important determinants of adult circulating HDL-C.

For triglycerides, we found that the combined detrimental effect of the 24 risk SNPs on triglyceride levels appears consistent over time for females from childhood through adulthood. For males however, both the cross sectional regression and IGG analyses suggest that the association between TGwGRS and fasting triglycerides levels increases linearly with age, becoming stronger on average than in females from around 25 years of age. These findings suggest that in adulthood, males at risk may respond less efficiently than females to lifestyle interventions targeting the reduction of fasting triglyceride levels

For lipids, it is largely unknown how a conventional age-varying ‘lifestyle-related’ risk factor such as adiposity modifies the genetic risk of developing abnormal lipid over the life course. This is a particularly relevant topic as identifying people whose risk is amplified by a combination of genetic and behavioral factors might facilitate interventions to prevent or delay the onset of cardiometabolic diseases. Δ BMIi, a proxy retrospective indicator of ‘adiposity trajectory’, was computed for each participant to summarize both the directionality and magnitude of change in their weight status-related lifestyle factors over their life course relative to the average change in the cohort. In our analyses, the change in BMI z-score from childhood to adulthood (Δ BMIi) predicted adult lipoprotein levels in 2011. This observation is consistent with the fact that adult BMI is important for many adult metabolic factors including lipids and that associations between lipids and BMI generally strengthened with age [55]. However, the effect of BMI z-score change since childhood on adult concentration is not modified by the wGRS for any of the lipoproteins, signifying that the detrimental consequences of an above average change in BMI since childhood on circulating lipoprotein levels does not appear to be exacerbated in adults that are genetically predisposed to high-risk lipoprotein profiles. However, this analysis is preliminary, and although it considers the overall direction of change if BMI z-score between childhood and adulthood, it does not fully account for the age-varying nature of BMI as a confounder and how it might interact with wGRS at different age-or developmental stages (childhood, adolescence, young adulthood). A study looking at the importance of the age at which obesity developed in associations between adult lipids and weight status, showed that although obese adults had adverse levels of lipoproteins, these levels did not vary with childhood weight status or with the age at the onset of obesity[56]. It remains unknown however if specific age of onset obesity modifies the effect of a genetic predisposition to averse lipoprotein levels, and whether primary prevention measures could be improved by specifically aiming at resolving obesity before a certain ‘critical’ age-window in individuals genetically at-risk to develop dyslipidemia, as has been shown for apparently healthy individuals [57].

The present work has a number of strengths and limitations. We studied a large, randomly selected cohort of men and women followed up at 8 occasions over the course of 30 years since early childhood. The extensive longitudinal lipoprotein phenotypic and genotypic data offered a rare opportunity for a refined analysis of the association between genetic risk and serum lipoprotein trajectories. The hierarchical mixed effect Individual Growth Curve modelling approach allowed us to comprehensively model between-individual changes in within individual outcome trajectories. The multivariable models of adult lipoprotein allowed estimating the ability of the polygenic GWAS-derived lipoprotein risk scores to predict adult lipoprotein concentration over other conventional childhood risk factors. Because the cohort is of European descent, our results are only generalizable to individuals with a similar ancestry. Loss to follow-up from the original cohort more often occurs for males, therefore the sex-specific time-averaged and time-dependent effect of multi-loci risk scores might be slightly biased. We also suggest that to validate causal inference, our findings should be tested for potential confounding effects of other variables such as additional adiposity indicators, which are known to correlate highly with lipoproteins and vary over time. However, additional adiposity phenotypes have not been collected at each study wave in the Cardiovascular Risk in Young Finns Study.

## Conclusions

This study demonstrates the significance of GWAS-derived genetic risk scores as predictors of lipoprotein levels at all ages. Additionally, we report for the first time an age-dependent effect of the 38 HDL-C risk SNPs on HDL-C and the 25 TG risk SNPs on TG levels among males, suggesting that the genetically-determined effects on these lipoproteins tends to change as a person ages. Further studies are now needed to characterise how this polygenic effect translates in terms of disease status prediction from childhood to adulthood.

## Supporting Information

S1 Appendix Step by step IGC model-building procedure, explicit parametrization of the IGC submodels and references on IGC modelling. (DOCX)

S2 Appendix Kernel density plots and quantile-quantile plots of LDL-C, HDL-C, and triglyceride concentrations (in mmol/L). (DOCX)

S1 Fig Flowchart of the IGC analyses used for modellng the lipid trajectories over the life course in the cohort. (JPG)

S1 File Original cohort and design of the Young Finns Study from 1980 through 2011 (Table A) and number of non-missing lipid measurements per study year in the subsample of the cohort participants considered in the present analyses (Table B). ^a, b^ Limitations in the sampling size in 1989 are due to physical examinations and blood tests collected only in one centre (Turku). In 1992, the limitation was due to economic constraints. * Maximum of participants in the current study was 2442 (i.e. participants successfully genotyped in 2001).
(DOCX)

S2 File Histograms of composite genetic risk scores (wGRSs) (Fig A) and of the number of available lipid observations per participants (Fig B). For each lipid, green colour in histogram A denotes the lower GRS quartile (i.e. the 25^th^ percentile); red colour, the upper GRS quartile (i.e. the 75^th^ percentile); and white colour, the interquartile range. (i.e. the 50% of the data lying between upper and lower quartile).
(DOCX)

S1 Table Estimation of interaction between polygenic lipoprotein risk scores and change in BMI z-score between adulthood (ages>30 years) and childhood (ages up to 18 years) on adult lipoprotein concentration prediction. *Abbreviations*: HDL-C, high-density lipoprotein cholesterol; LDL-C, low-density lipoprotein cholesterol, wGRS-HDL; *HDLwGRS3* continuous combined genetic risk score for HDL (38 SNPs); wGRS-HDL, *LDLwGRS14*, *continuous* combined genetic risk score for LDL-C (14 SNPs); wGRS-TG, *TGwGRS24*, *continuous* combined genetic risk score for triglycerides (24 SNPs); adult.age, age of participants at latest adult follow up (2001, 2007 or 2011*)*,Δ BMI, change in BMI z-score between childhood and adulthood as described in Methods section. * For HDL-C and LDL-C, the effect of wGRSs is in mmol/L per 1-sd change in wGRS, and the effect of Δ BMI is in mmol/L per 1-sd change in Δ BMI. For triglycerides, the effect of wGRSs is in odds ratio per 1-sd change in wGRS, and the effect of Δ BMI is in odds ratios per 1-sd change in Δ BMI.
(PDF)

S2 Table List of lipid-associated SNPs used to generate the genetic risk scores for HDL-C and LDL-C in the YF population (reported are the SNPs reference number, risk allele for the SNP, GWAS-derived effect size in md.dl^-1^). The gene name listed under ‘locus’ is either plausible biological candidate gene in the locus or the nearest annotate gene to the SNP. ‘chr’ denote chromosome. ‘Freq (%)’ denotes the risk allele frequency in the YF population, Adapted from Teslovitch et al. (2010)[23] and Tikkanen et al. (2011) [29].
(DOCX)

S3 Table Childhood and adult cutoffs used to define ‘normal’ and ‘high-risk’ serum lipid levels (in mmol/L). Childhood cutoffs apply to participants 19 years and younger. Chosen cutoffs are based on NCEP adult-panel treatment guidelines, NECP pediatric and adolescent guidelines and AAP and AHA pediatric guidelines (see methods).
(DOCX)

S4 Table Sex-specific associations between categorical genetic risk score and lipid levels at selected ages (3 years, 15 years, 24 years and 45 or 46 years). Effect sizes β are in mmol/L for the number of risk allele differences between high and low wGRSs. Reported at each age are: Int (SE), the average lipid level in mmol/L (SE) in the high risk score group; β (SE), the difference in average lipid level in mmol/L for subjects in the low risk score group, the p-value for each cross-sectional association and the number of non-missing serum lipid observations considered in each regression (Nobs). (*: For each sex, the number of risk alleles difference between high and low wGRS groups are calculated as: median (Number of alleles in the High wGRS)–median ((Number of alleles in the Low wGRS)).
(DOCX)
