# Supplementary material for: The Combined Effect of Common Genetic Risk Variants on Circulating Lipoproteins Is Evident in Childhood: A Longitudinal Analysis of the Cardiovascular Risk in Young Finns Study
Source: PLoS One. 2016 Jan 5;11(1):e0146081. doi: 10.1371/journal.pone.0146081 (PMC4701181; doi:10.1371/journal.pone.0146081)
Supplement: S1 Appendix — (DOCX) [file pone.0146081.s001.docx]

**Step by Step protocol for IGC modeling**

**Data exploration**

Prior all IGC analyses, plotting raw data trajectories of a number of randomly selected YF participants suggest variability in individuals’ initial lipid levels and variability in how individuals’ lipid levels change as they age. We explored the shape of each lipid trajectories across the life course using individual empirical growth plots and generalized additive mixed models (GAMMs). GAMM is a well-suited modelling framework for uncovering potentially non-linear trends in correlated datasets. This approach allows flexible specification of regression splines to represent the functional relationships between a response variable and a temporal covariate that can be continuous or discrete (i.e. age) using smooth functions of the covariate (i.e. age in our case). The fitting of GAMMS can also suggests plausible polynomial models to describe the pattern of change in lipid model (i.e. typically, you choose the model order by the number of bends you need in the predicted GAM trajectory (number of inflection points)).

*Note:* To conduct IGC analysis, 3 or more waves of data collection are required; having 2 or less will limit the ability to identify shape of individual growth trajectories and to distinguish true growth from measurement error. Theoretically, it is possible to test for linear growth with a minimum of 3 data waves per subject, quadratic growth with 4 waves minimum, and so one. In our study sample there is a maximum of 8 waves of data for each YF participant so we could theoretically fit an age polynomial up to the order 7. However, in practice, because participants had 5.31 lipid observations each in average in our study sample, the highest order age polynomial term we consider to describe individual lipid profiles is age^4^ (i.e. quartic age growth).

**Individual Growth Curve modelling (IGC analysis)**

IGC modelling is a multilevel regression technique used for exploring longitudinal data on individuals over time. It allows investigating two levels of variability of the response variable: within and between subjects. In longitudinal data, measurements made on the same individual are correlated and it is this dependency that leads to the inadequacy of simple estimation procedures based on ordinary least squares. Observations taken over time are nested within subjects drawn from some population of interest giving a two-level hierarchical structure. The variation of responses within subjects over time is at the lowest level (level 1) and the variation of the underlying mean responses between subjects is at level2 [1]. Growth curve analysis allows to model and quantify change over time both at group-level (level 2) and individual-level (level 1). Sometimes in longitudinal data the interest lies as much in the covariance matrix estimates as in the average growth parameters. IGC allows examining both. In our case, the time-relevant predictor is age and the predictor of interest is genetic risk scores (wGRSs) as we want to determine if wGRSs modify the trajectories of blood lipids across the lifetime. Because previous research reports different lipid trajectories profiles in male and females across the lifecourse, we also want to test how ‘sex’ modifies the lipid trajectories. In practice, IGC modelling consist in sequentially testing a number of models as follows:

***Step 1) Unconditional means (UM) model***

The UM model is the simplest multilevel model contains no predictor (i.e. this is often viewed as a one-way ANOVA model with subject-level random intercept). It serves as a baseline model to examine individual variation in the outcome variable without regard to age. This model assesses (1) the grand mean of the outcome variable and (2) the amount of outcome variation that exists in intra- and interindividual levels. It allows computing the ICC ( intraclass correlation coefficient), which describes the amount of variance in the outcome that is attributed to differences between individuals. The ICC evaluates the necessity of modelling the nested data structure. For LDL-C, the UM model can be written as:

*Level 1: LDL_ij_ =* b_oi_ + e_ij_

Level 2 : b_oi=_ β_0_ + u_0i_

Where e_ij_ ~_iid_ *N* ( 0_,_ σ^2^) and u_0i_ ~_iid_ *N* ( 0_,_ τ^2^)

Example of UM model for LDL-C in R notation ( nlme package):

*UMmodel<- lme (LDL-C ~1, random= 1|subject, method = "ML", na.action = na.omit)*

For example, when applying the UM model to LDL-C data, we calculated an ICC of 0.67, suggesting that about 67% of the total variation in the LDL-C level was due to inter-individual difference: it argues strongly in favor of fitting a multilevel model.

***Step 2) Potential unconditional growth (UG) models***

The next step is to determine the Unconditional growth model (UG model), which in our case is a model where the response variable (i.e. lipid level) is modelled as a function of age, and where participants IDs are used as a random effect (i.e. random intercept). This is the level1 model, describing how each person lipid levels change overtime. In UG models, subjects are collapsed across all possible grouping variables of interests (i.e. sex, risk factors …), and age (or any other relevant time variable) is expressed as a deviation from average value in the cohort (i.e. centering the time-related variable will reduce multicollinearity between polynomial terms if a higher order polynomial is needed to fit the data).

It is common practice to always fit an Unconditional Linear UG Model, even when data exploration suggests non-linear response overtime. The linear UG model is used as a baseline to compare curvilinear UG models for the response overtime. Orthogonal polynomials are a possible choice to model non-linear response variables by IGC analysis. Unlike natural polynomials, orthogonal polynomials terms have the advantage to be interdependent (no multicollinearity between age, age^2^, age^3^…). When using orthogonal polynomials, the intercept term of the model will reflect the average overall curve height, rather than the height at the left edge of the time window, but higher order parameters have difficult interpretation. They are usually preferred when the main interest lies in prediction.

In our application, we used natural polynomial to capture the curvature of the lipid profiles because we were interested in differences at the very beginning of the time window (i.e. age was centered around the youngest age in the cohort (age 3)). Also our interest lies more in the pattern of change in the response overtime, rather than in which time component is more important. Therefore was more desirable to interpret the output of the linear mixed effect models on the original time-scales. Additionally collinearity between covariates does not tend to affect the quality of parameter estimation to a notable extend, so we opted for centering of the age terms (around 3 years old) rather than using orthogonal polynomials as recommended by Liu and Engel 2015[2]

*Example : Potential UG models for LDL-C:*

*# linear UG model (with a random intercept only):*

*Level 1: LDL_ij_ =* b_oi_ + b_1_.Age*_ij_* +e_ij_ # Addition of age as a predictor at level 1

Level 2 : b_oi=_ β_0_ + u_0i_

Where e_ij_ ~_iid_ *N* ( 0_,_ σ^2^) and u_0i_~_iid_ *N* ( 0_,_ τ^2^)

In lme syntax, the linear UG can be written as:

*Linear. UG<- lme (LDL-C ~ age, random= 1|subject, method = "ML", correlation, na.action = na.omit)*

However, the fitting of a GAM models in the data exploration suggested that the lipid responses are curvilinear, probably modelled with a cubic or 4^th^ order polynomial polynomial. So we also fit the following potential UG models:

*# quadratic UG model (with a random intercept):*

*Level 1: LDL_ij_ =* b_oi_ + b_1_.Age*_ij_* + b_2_.Age^2^*_ij_* +e_ij_ # Addition of age-square as a predictor

Level 2 : b_oi=_ β_0_ + u_0i_

Where e_ij_ ~_iid_ *N* ( 0_,_ σ^2^) and u_0i_ ~ _iid_ *N* ( 0_,_ τ^2^) with e_ij_ and u_0i_ are independent for all *i* and *j*.

In R notation, this model is written:

*Quadratic. UG<- lme (LDL-C ~ age+* *I(age^2)+ , random= 1|subject, method = "ML", correlation, na.action = na.omit)*

Similarly, we can define mode complex level 1 submodels that include cubic or quartic growth predictors:

*# cubic UG model (with a random intercept)*

*cubic. UG<- lme (LDL-C ~ age+* *I(age^2)+ I(age^3) , random= 1|subject, method = "ML", correlation, na.action = na.omit)*

*# quartic UG model (with a random intercept)*

*quartic. UG<- lme (LDL-C ~ age+* *I(age^2)+ I(age^3)+ I(age^4)+ , random= 1|subject, method = "ML", correlation, na.action = na.omit)*

***Unconditional Growth Model Comparisons:***

When comparing increasingly complex submodels throughout the IGC analyses, the improvement in model fit is assessed by likelihood ratio test or by looking at AIC/BIC criteria. We determine the best fitting UG model among these candidate level 1 submodels using both.

1*) likelihood ratio tests (LRT):* can only be used **to**  compare models are nested within one another (i.e. this is done by computing -2 times the difference between the two models residual log likelihoods (-2RLL), and comparing it to the χ^2^ distribution with degrees of freedom equal to the difference in the number of parameters for the two models). Models are preferred where the -2RLL is smaller.

2) *Akaike’s Information Criterion (AIC)* is also useful as it is valid to compare even non-nested models (i.e. model with the smallest AIC is preferred).

It is important that the candidate level 1 models are fit using full maximum likelihood (method="ML" argument in R), so that the LR tests can be used to evaluate the elimination of either fixed or random effects. The adequate UG model will be the retained as Baseline model for the next steps in the IGC analysis.

*Example:*

*anova(UMmodel ,linear.UG, quadratic.UG, cubic.UG, quartic.UG)*

*AIC(UMmodel ,linear.UG, quadratic.UG, cubic.UG, quartic.UG)*

*At this stage, AIC criteria and LR tests showed the cubic growth model with variance in individual intercept was best at capturing the intra-individual growth of LDL-C overtime.*

*So far, LDL-C UG model, with age_ij_ centered around 3 years old, can be written:*

*Level 1 : Y_ij_=b_oi_ +b_1_(age_ij_) + b_2_(age_ij_)^2^ + b_3_ (age_ij_)^3^ + e_ij_*

Level 2 *: b_oi_*_=_ β_0_ + u_0i_

*With e_ij_,the amount the response on occasion j deviates from person i's true change trajectory*

**Step 3) Model the random effect structure** (i.e.subject-level intercept and slope variability)

Once the best UG model has been determined, it is possible to explicitly model the random part of this model. Indeed, a potential limitation of the UG model chosen above for LDL-C (i.e. cubic growth model with random intercept) is that is assumes that the relationship between time and LDL-C is constant for all individuals. An alternative model could be one where unobserved individual characteristics would also explain variation in trajectory parameters This means expanding the level 2 model by adding random terms for age-, age^2-^, and age^3^ slopes.

There are diverging views in the literature regarding the specific modelling of random effects in IGC analysis. Barr et al. [3], do not advocate model selection of the random growth parameters, suggesting that UG models should include all random effects that are licensed by the design (i.e. all the ones that could potentially vary across participants. This is sometimes referred to as the ‘ beyond optimal random effect structure’, where all age terms in the fixed effect part of the growth model also appear in the random part of the model.  The reason for allowing all possible slopes to vary across individual is that even when a random effect does not improve model fit, it can still affect the fixed effect estimates and excluding it can elevate the false positive rate. However, estimating random effects is “expensive” in terms of the number of observation required, and estimating too many random effects can lead to computational difficulty (i.e. model failing to converge). Mirman et al.[4,5] usually only include participant random effects on all time terms up to the cubic, as terms beyond this tends to capture less-relevant effects in the tails. Others, including Pinheiro et al. [6], recommend testing the significance of all possible random terms when higher-level trends are significant, to determine whether allowing the slopes of higher-level variable to vary randomly will improve model fit (i.e. test that a quadratic or cubic relationship varies in strength among individuals).

Our approach was (when technically possible) was to test sequentially if each additional random parameter (i.e. random intercept, random linear slope, random quadratic slope…) improved the fit of the unconditional UG models using LR tests and AIC, as in Zuur et al. and Bliese [7,8]. This is done in our model by adding, in order of increasing complexity, the linear, quadratic, and cubic age terms as random effects in the cubic UG growth model. If one wants to select ad once of the optimal fixed effect and random effect structure, it is best to use ML estimation rather than REML as in [6]. Here because we have determine that the best model was the cubic UG mode, we can used the restricted maximum likelihood “REML” estimation method, so that LR tests evaluate the elimination of random effects only.

Once the optimal random effect structure has been chosen for the UG model, one can visualise the variance covariance matrix of the random effects at level 1 (i.e for lme objects in R, one can access random effect’s variance and correlation estimates using the VarCorr () argument). It will reveal, for example, whether within-individual lipid levels at baseline (intercept) are correlated with linear change in lipid across the life course. As is common with random effects, we apply no constraint on the variance-covariances of the random effect matrix (i.e. unstructured G-matrix)

*Example : with age centered around 3 years old, LDL-C UG sub-models with slope variability include:*

*# cubic UG model (with a random intercept) == best level 1 model as determined by LR test above*

*cubic. UG<- lme (LDL-C ~ age+* *I(age^2)+ I(age^3) , random= 1|subject, method = "REML", correlation, na.action = na.omit)*

*# cubic UG model with a random intercept and linear age slope: (2X2 unstructured variance covariance matrix):* At this point we have three variance components: σ^2^_,_ τ_0_^2^ and τ_1_^2^ . The correlation between intercept and linear slope is given by $\tau_{01}$.

*Level 1 : Y_ij_=b_oi_ +b_1i_(age_ij_) + b_2_(age_ij_)^2^ + b_3_ (age_ij_)^3^ + e_ij_*

Level 2 *: b_oi_*= β_0_ + u_0i_

*b_1i_* = β_1_+ u_1i_

Where in addition : e_ij_ ~_iid_ *N* ( 0_,_ σ^2^) and u_i_= $\left[ \begin{aligned} u_{oi} \\ u_{1i} \end{aligned} \right]$~_iid_ *N* $\left( \left[ \begin{matrix} o \\ o \end{matrix} \right] \right.\left. ,\left[ \begin{matrix} \tau_{o}^{2} & \tau_{01} \\ \tau_{01} & \tau_{1}^{2} \end{matrix} \right] \right)$

In R notation :

*cubic. UG. 1<- lme (LDL-C ~ age+* *I(age^2)+ I(age^3) , random= 1+age|subject, method = "REML", correlation, na.action = na.omit)*

*It is also possible to add additional variance components at level 2 (i.e. random quadratic, and cubic slopes):*

*# cubic UG with a random intercept and random linear and quadratic slopes: (3X3 unstructured variance covariance matrix)*

*Level 1 : Y_ij_=b_oi_ +b_1i_(age_ij_) + b_2i_(age_ij_)^2^ + b_3_ (age_ij_)^3^ + e_ij_*

Level 2 *: b_oi_*= β_0_ + u_0i_

*b_1i_* = β_1_+ u_1i_

*b_2i_*= β_2_ + u_2i_

Where in addition : e_ij_ ~_iid_ *N* ( 0_,_ σ^2^), and u_i_= $\left[ \begin{matrix} u_{0i} \\ u_{1i} \\ u_{2i} \end{matrix} \right]\sim\text{iid}\left( \left[ \begin{matrix} o \\ o \\ o \end{matrix} \right],\left[ \begin{matrix} \tau_{O}^{2} & \tau_{01} & \tau_{02} \\ \tau_{01} & \tau_{1}^{2} & \tau_{12} \\ \tau_{02} & \tau_{12} & \tau_{2}^{2} \end{matrix} \right] \right)$

*In R notation, this can be written as:*

*cubic. UG. 2<- lme (LDL-C ~ age+* *I(age^2)+ I(age^3) , random= 1+age+ I(age^2)|subject, method = "REML", correlation, na.action = na.omit)*

For a level 1 model with a 3^rd^ order polynomial, the maximum possible random parameters for the variance covariance matrix is 4x4:

*# cubic UG with a random intercept and random linear, quadratic and cubic slopes:*

*cubic. UG. 3<- lme (LDL-C ~ age+* *I(age^2)+ I(age^3) , random= 1+age+ I(age^2) + I(age^3)|subject, method = "REML", correlation, na.action = na.omit)*

To determine the best random effect structure, the increasingly complex submodels (in terms of additional variance and covariance components for the random effects) are then compared using AIC and/or LR tests.

mod.aic<-sapply(list(cubic. UG, cubic. UG. 1, cubic. UG. 2, cubic. UG. 3), AIC)

LR-test<-sapply(list(cubic. UG, cubic. UG. 1, cubic. UG. 2, cubic. UG. 3), anova)

*Example:*

*In the case of the LDL-C UG model, all 4-candidate structures for random effects could be estimated with no convergence issue. The cubic growth model with a random intercept, linear and quadratic slopes was the best fit to the data as determined by LR test and AIC criteria (with a 3 x 3 unstructured variance covariance matrix for the random effects). In this model τ_0_^2^ refer to the variability in the intercepts of the subject-specific regression lines about the intercept of population-averaged line, τ_1_^2^ is the variability of the individual slopes about the population linear slope, and τ_2_^2^ , the variability of the individual slopes about the population line quadratic slope. σ^2^ is the average variability of participants LDL-C values about their own individual regression line*

**Step 4) Model residual error structure**

This is very important to scrutinize level 1 error structure especially with unequally spaced and unbalanced data, because estimated variances of the parameter estimates are likely to be biased and affect the precision of estimating the appropriate model.

The purpose of testing different error covariance matrices is to describe how UG model the error is distributed. It is likely that lipid measurements taken temporally close to each other will be more strongly related than those temporally far apart (i.e. autocorrelation). It is also likely that lipid levels will tend to become either more variable over time or less variable over time as participants age (i.e. heteroscedasticity). UG models need to be tested for both autocorrelation and heteroscedasticity. The Models fitted to select the optimal error structure are also fitted using ML (maximum likelihood) as in [8].

***A/ Residual correlation structure:***

In the present study, three commonly examined types of error covariance structures were tested for the UG models: unstructured, compound symmetric, and first-order autoregressive variance covariance of the residuals’ matrix (Pinheiro and bates 2013[6]):

1) *Unstructured (UN)*, often offers the best fit in longitudinal data as it requires no assumption in the error structure. The variance is not assumed constant between different ages and the correlations between observation times can all be different (estimation can be problematic as many parameters as number of timepoints -1 need to be estimated). In lme notation such a residual correlation structure is defined as: correlation = corSymm(form=~1|subject) within the model formulation.

2) *Compound Symmetry (CS*), implies that the variance and correlation between each pair of observations are constant across time points (i.e. correlation = corCompSymm (form=~1|subject))

3) *Continuous First-Order Autoregressive (AR1)*, in which the variance is assumed to be heterogeneous and the correlations between the two adjacent time points decline across measurement occasions (i.e. correlation = corCAR1(form = ~ age| subject).

*Example:*

*In our LDL-C UG model example, the LR-test showed that a model with an AR1 error structured fit the data better, so it will be retained when introducing level 2-predictors in the model.*

***B/ Residual heteroscedasticity:***

Examining the variance of lipid concentrations at each age can give an idea of variance homogeneity. For each lipid UG model we tested whether the error variance decreased or increased over ages (i.e. weights=VarFixed(form=~age) or weights=VarExp(form=~age) arguments in the lme() call respectively).

*Example: For all lipid UG models, residual variance did not exhibit significant increasing or decreasing trends overtime so that subsequent models were not adjusted for heteroscedasticity.*

**Step 5) The conditional growth (CG) sub-models**

Once the fixed and random components of the unconditional growth model have been determined, we can introduce independent predictors (time-varying or time invariant) to the lipid growth model: it becomes the conditional growth model. This is the level 2 model (i.e. between-person model) describing how the individual growth differs across individuals. The CG model represents the relationship between level 1 growth parameters (intercepts, linear, quadratic and cubic slopes) and time-invariant characteristics of the individuals (e.g. in our application, wGRS group (‘low’, ‘high’, “mid’) or continuous wGRSs ; and sex). The Level 2 model captures whether the rate of change varies across individuals in a systematic way. The growth parameters (i.e., the within-subjects intercepts and slopes) of Level 1 are the outcome variables to be predicted by the between-subjects variables at Level 2.The CG model allows to answer questions such as: are there differences between genetic risk group (High vs low) in the lipid change overtime? How does a 1 SD increase in wGRS will impact the average lipid trajectory as a person age?

1. ***Add sex as level-2 predictor in the model:***

table(LDLgrsdata$sex)

Female Male

5192 4544

Sex is a factor variable in R. By default dummy variables have been constructed to contrast temperate and male with female participants:

*contrasts(LDLgrsdata$sex)*

*Male*

*Female 0*

*Male 1*

 Now we can model how “sex’ modifies the basic level-1 relationship between LFL-C and age. It can potentially affect: intercept, the slopes, or both? Similarly as what is described above, we can sequentially test the 3 models:

1. *Does sex affect the population LDL-C intercept (LDL-C levels at age 3) ?*

# Baseline level 1 model for comparison:

*cubic. UG. 2<- lme (LDL-C ~ age+* *I(age^2)+ I(age^3) , random= 1+age+ I(age^2)|subject, method = "ML", correlation, na.action = na.omit)*

model3<-lme(LDL-C~I(*LDL-C ~ age+* *I(age^2)+ I(age^3)* +sex*, random= 1+age+ I(age^2) |subject, method = "ML", correlation, na.action = na.omit)*

anova(model3, *cubic. UG. 2*)# LR-test, P-val= 0.45

# Note: because ‘sex’ is a categorical variable with only two categories, the Wald tests shown in the summary output of model3 will provide a test for the significance of “sex” the intercept (it won’t be different from the LR-test). However it is good practice to use likelihood ratio test anyway, as for variable with more than 2 levels, it is necessary to apply LR-test to obtain significance of the variable.

It is possible further quantify the relationship between sex and the intercept of the LDL-C model by computing a pseudo-R^2^ statistic:


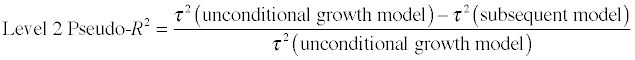


as.numeric(VarCorr(*cubic. UG. 2*)[2,1]) - as.numeric(VarCorr(model3)[2,1])) / as.numeric(VarCorr(*cubic. UG. 2*)[2,1])= 0.04

So only 3% of the individual-level variation in the intercept (i.e. LDL-C levels at age 3) is is explained by “sex”. This is consistent with the fact that adding “sex” to the level-2 equation for the intercept did not significantly improve the model (LR –test, p-value=0.45)

1. *Does sex affect the population linear slope?*

Similarly to what has been shown above, we can compute:

model4<-lme(LDL-C~I(lntemp-~ age+ I(age^2)+ I(age^3) +sex:age, random= 1+age+ I(age^2) |subject, method = "ML", correlation, na.action = na.omit)

anova(model4, *cubic. UG. 2*)

The likelihood ratio test finds the effect of climate on the slope to be statistically significant. This is confirmed by looking at the AIC. We can also quantify the effect with a pseudo-R^2^ statistic (i.e. this time it compares the change in $\tau_{1}^{2}$ between the current model and random slopes and intercepts model (cubic.UG.2 model)):

(as.numeric(VarCorr(cubic. UG. 2)[2,1]) - as.numeric(VarCorr(model3)[2,1])) / as.numeric(VarCorr(cubic. UG. 2)[2,1])= 0.04

*3- Does sex affects the all population trajectory parameters?*

Following the same approach, we use LR-T test, AIC and level 2 pseudo-R^2^ to test sequentially the effect of sex on every trajectory parameter:

The most complex model, where sex affects all trajectory parameters, can be written as:

*Level 1 : Y_ij_=b_oi_ +b_1i_(age_ij_) + b_2i_(age_ij_)^2^ + b_3_ (age_ij_)^3^ + e_ij_*

Level 2 *: b_oi_*= β_0_ + β_01_ (sex_i_) +u_0i_

*b_1i_* = β_1_+ β_11_ (sex_i_) +u_1i_

*b_2i_*= β_2_ + β_21_ (sex_i_) + u_2i_

In R notation:

model5<-lme(LDL-C~I(lntemp-*~ (age+* *I(age^2)+ I(age^3)* )*sex*, random= 1+age+ I(age^2) |subject, method = "ML", correlation, na.action = na.omit*

anova(model5, *cubic. UG. 2*)

AIC and LR tests showed that model 5 was the best level 2 model for LDL-C at this stage.

1. ***Add wGRS risk group as level-2 predictor in the model:***

Similarly to what has been demonstrated above for the ‘ sex’ variable above, we test how wGRS group influences individual growth parameters of LDL-C. In this model contrasts where chosen to compare low and mid genetic risk groups with high genetic risk category:

*contrasts(LDLgrsdata$GRS)*

*low mid*

*high 0 0*

*low 1 0*

*mid 0 1*

Similarly to what we have shown for sex, we test the effect of GRS group on LDL-C intercept and age slopes using LR-t test and AIC.

*Example: Hypothetic level-2 model for LDL-C with wGRS group affecting all trajectory parameters:*

Level 1 : Y_ij_=b_oi_ +b_1i_(age_ij_) + b_2i_(age_ij_)^2^ + b_3_ (age_ij_)^3^ + e_ij_

Level 2:

b_oi_ =β_00 +_ β_02_ (wGRS_i_) + ζ_0i_ # individual intercept

b_1i_ =β_10 +_ β_12_ (wGRS_i_) + ζ_1i_ # individual linear slope

b_2i_ =β_20 +_ β_22_ (wGRS_i_) + ζ_2i_ # individual quadratic slope

b_3_ =β_30 +_ β_31_ (wGRS_i_)

1. ***Test the combined effects of sex and wGRSs groups and their interaction as level 2 predictors: Final IGC model***

*At last, the final IGC model is the one where all level-2 predictors and their cross-products are added in the model. It allows to test the effect of the dichotomous genetic risk score category, sex, and their interaction (wGRS group by sex) on the shape of average LDL-C growth trajectories in the cohort. It is used to explore any group differences in change over time. The corresponding final IGC model can be written as:*

*LDLserum.lme <- lme(LDLserum ~ sex*(age+I(age^2)+I(age^3))+ LDLwGRS14*(age+I(age^2)+I(age^3)) + (sex* LDLwGRS14)* (age+I(age^2)+I(age^3)) , random=list(Patients=~1+age+ I(age^2)),*

*method = "ML",correlation = corCAR1(form = ~ age| subject),data = LDLgrsdata,na.action = na.omit,control=lmeControl(opt="optim"))*

Final IGC model, with level-2 predictors sex and wGRSs groups and their crossproducts:

Level 1 : Y_ij_=b_oi_ +b_1i_(age_ij_) + b_2i_(age_ij_)^2^ + b_3_ (age_ij_)^3^ + e_ij_

Level 2:

b_oi_ =β_00 +_ β_01_ (sex_i_) _+_ β_02_ (wGRS_i_) + β_03_ (sex_i_* wGRS_i_) + u_0i_ # individual intercept

b_1i_ =β_10 +_ β_11_ (sex_i_) _+_ β_12_ (wGRS_i_) + β_13_ (sex_i_* wGRS_i_) + u_1i_ # individual linear slope

b_2i_ =β_20 +_ β_21_ (sex_i_) _+_ β_22_ (wGRS_i_) + β_23_ (sex_i_* wGRS_i_) + u_2i_ # individual quadratic slope

b_3_=β_30 +_ β_31_ (sex_i_) _+_ β_32_ (wGRS_i_) + β_33_ (sex_i_* wGRS_i_) # population average cubic slope

- - β_00_ = population level average intercept (fixed effect) and β_10_ = population average linear slope, β_20=_=population average quadratic slope, b3=population average quadratic slope
  - β_01_, β_02_, and β_03_: three predictors of intercept variability, respectively for sex, wGRS and sex*wGRS interaction.
  - u_0i ,_   u_1i_ , u_2i :_ the amount person i's intercept, linear slope, and quadratic slopes deviate from the population average parameters
  - e_ij_ : the amount the response on occasion j deviates from person i's true change trajectory, and e_ij_ follows a AR1 structure.

Significance of each predictor in the the final UG model is assessed by t-statistics for the parameter *(i.e. defined as the ratio of parameter estimate and SE).* Reported in the Tables 2 and 3 are the p-values provided in the LME () output in R.

*Example: Parameter estimates presented in Table 2 and 3 for LDL-C correspond to β_02_, β_12_ β_22_ and β_32_ parameters of the final IGC models, as all sex* wGRS interaction terms (i.e. β_03,_ β_13,_ β_23_ and β33) were unsignificant in the final model. The fixed estimates from the final IGC model output can be used to compute the slope estimates for the 6 relevant groups (i.e. male –High risk, male-mid risk, male -low risk, female-high risk, female-mid risk and female low-risk) (Figure 1).*

**IGC modelling vs. ANOVA?**

The specification of an IGC model is different to an ANOVA model: Although individual growth curve models can treat age as continuous and variable across subjects, and model change in outcome at the individual level, the ANOVA model approach focuses on growth curves at the aggregate level. ANOVA assumes that all individual are measured at the same time-point for any given wave of data collection. One of the advantages of IGC models over ANOVA, is that they can treat age as a truly continuous variable. ANOVA also doesn’t model individual variability in rates of change in the outcome overtime

**Adjustment for confounding effects:**

**1- Birth cohort effect**

To minimize potential confounding effects, we considered a birth cohort effect on the age-related changes in lipids in the YF cohort for each sex. Before introducing genetic risk scores (wGRSs) as predictors of lipid trajectories, we thus assessed the effect of ‘Year of birth’ (yob) on each lipid’s unconditional growth model. This was done by adding the variable ’Year of birth” and its interaction terms with age-related terms to each sex-specific lipid trajectory model (i.e. for female LDL-C model, for example: age, age^2^, age^3^, yob, yob*age, yob*age^2^ and yob*age^3^).’yob’ was used as a categorical variable with 6 levels, for the 6 possible birth cohorts represented in our data sample (i.e. participants born in 1977, 1974, 1971, 1968, 1965, and 1962 respectively). Similarly to what has been described above for sex and wGRSs group, LR-T test and AIC were used to test the significance of a birthcohort effect on the lipids’ trajectory parameters.

***2-Period effect***

Similarly, before considering the predictive effect of the genetic risk scores on the lipid trajectories, we considered potential differences in lipid measurements between examinations and/or secular ‘period effect’ in lipids over the 31 years of follow-up. To test for this, we introduced calendar ‘year’ (centered around 1980 (baseline)) in the sex-specific lipid unconditional growth models (i.e. for LDL-C the model included age, age^2^, age^3^, year, year*age, year*age^2^ and year*age^3^).

*Example:*

*In all sex-specific analyses of lipid profiles, we found no birth cohort effect. However, in each case, we found significant secular trends (see Results). For this reason, all UG models were adjusted for ‘year’ before introducing subject-level trajectory predictors (i.e. wGRSs). For example, the final IGC model for LDL-C can be written as:*

*LDLserumlme <- lme(LDLserum ~ sex*(age+I(age^2)+I(age^3)))+ LDLwGRS14*(age+I(age^2)+I(age^3))) + (sex* LDLwGRS14)* (age+I(age^2)+I(age^3))) +****as.numeric(year),*** *, random=list(Patients=~1+age+ I(age^2)),*

*method = "ML",correlation = corCAR1(form = ~ age| subject),data = LDLgrsdata,na.action = na.omit,control=lmeControl(opt="optim"))*

.

**Final model check and validation**

Each final IGC model was checked using graphic model validation tools for mixed-models described in Zuur et al [9].

**References on IGC analysis:**

1. Singer JD, Willett JB (2003) Applied Longitudinal Data Analysis: Modeling change and event occurence. New York: Oxford University Press.

2. Liu X, Engel CE (2015) Methods and Applications of Longitudinal Data Analysis: Elsevier Science Publishing Company Incorporated.

3. Barr DJ, Levy R, Scheepers C, Tily HJ (2013) Random effects structure for confirmatory hypothesis testing: Keep it maximal. J Mem Lang 68.

4. Mirman D (2014) Growth Curve Analysis and Visualization Using R. : Chapman and Hall / CRC.

5. Mirman D, Dixon JA, Magnuson JS (2008) Statistical and computational models of the visual world paradigm: Growth curves and individual differences. J Mem Lang 59: 475-494.

6. Pinheiro J, Bates D, DebRoy S, Sarkar Dat (2013) Linear and Nonlinear Mixed Effects Models. R package version 3.1-110. R Development Core Team (2013).

7. Bliese PD (2009) Multilevel modeling in R (2.3) a brief introduction to R, the multilevel package and the nlme package.

8. Zuur A, Ieno EN, Walker N, Saveliev AA, Smith GM (2009) Mixed Effects Models and Extensions in Ecology with R: Springer.

9. Zuur AF, Ieno EN, Elphick CS (2010) A protocol for data exploration to avoid common statistical problems. Methods in Ecology and Evolution 1: 3-14.
