# Supplementary material for: The Combined Effect of Common Genetic Risk Variants on Circulating Lipoproteins Is Evident in Childhood: A Longitudinal Analysis of the Cardiovascular Risk in Young Finns Study
Source: PLoS One. 2016 Jan 5;11(1):e0146081. doi: 10.1371/journal.pone.0146081 (PMC4701181; doi:10.1371/journal.pone.0146081)

**S3 Appendix**

**LDL-C levels (mmol/L):**


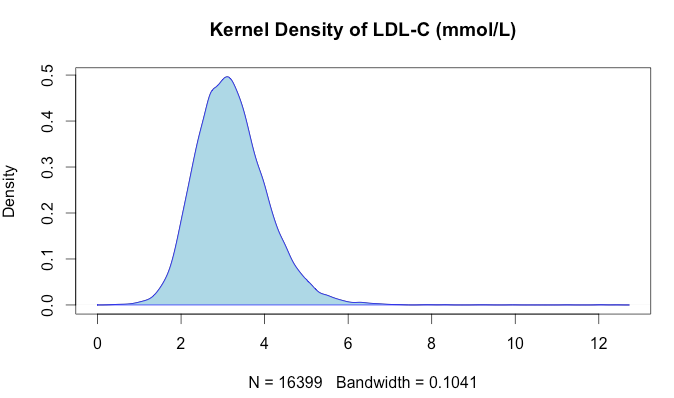


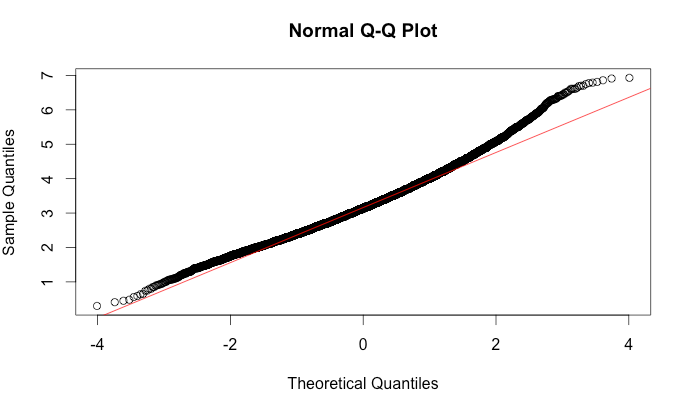


**HDL-C levels (mmol/L)**:


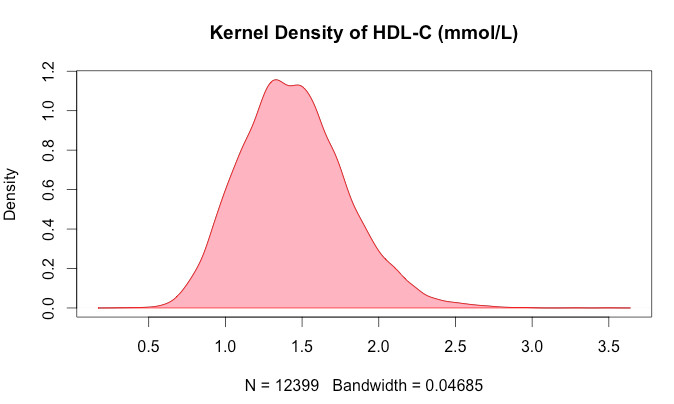


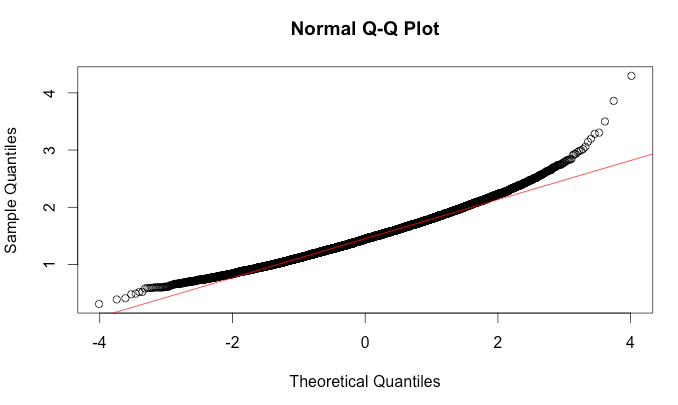


**Triglycerides levels (mmol/L):**

Before log transformation:


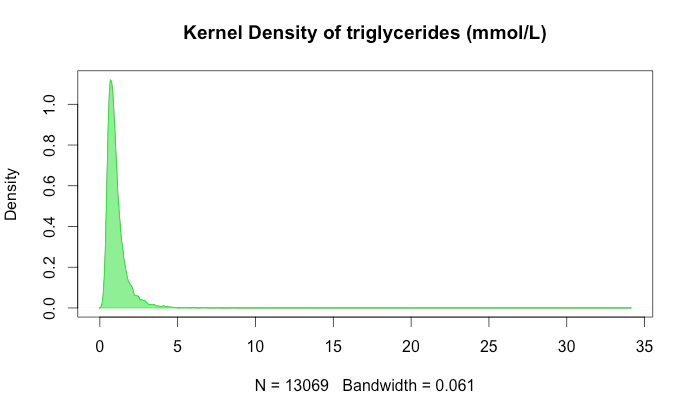


QQ plot of triglycerides distribution after log transformation:


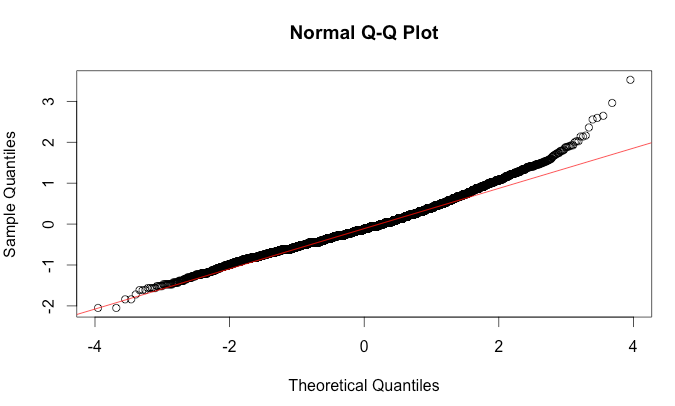

Supplement: S2 Appendix — (DOCX) [file pone.0146081.s002.docx]
