# Supplementary figures and images for: The Combined Effect of Common Genetic Risk Variants on Circulating Lipoproteins Is Evident in Childhood: A Longitudinal Analysis of the Cardiovascular Risk in Young Finns Study
Source: PLoS One. 2016 Jan 5;11(1):e0146081. doi: 10.1371/journal.pone.0146081 (PMC4701181; doi:10.1371/journal.pone.0146081)

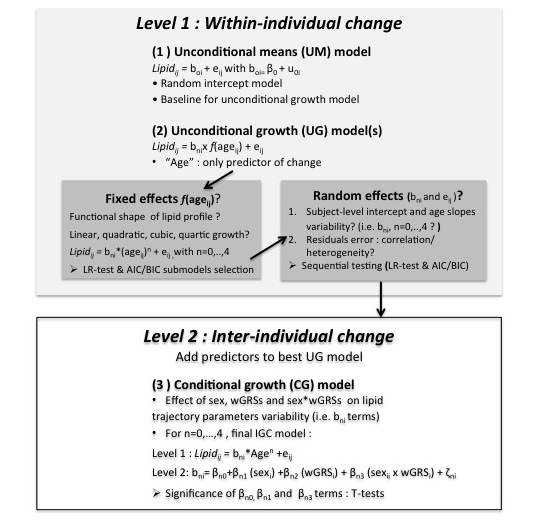

Supplement: S1 Fig — (JPG) [file pone.0146081.s003.jpg]

**S2 File**

**Figure A**

**Figure B**


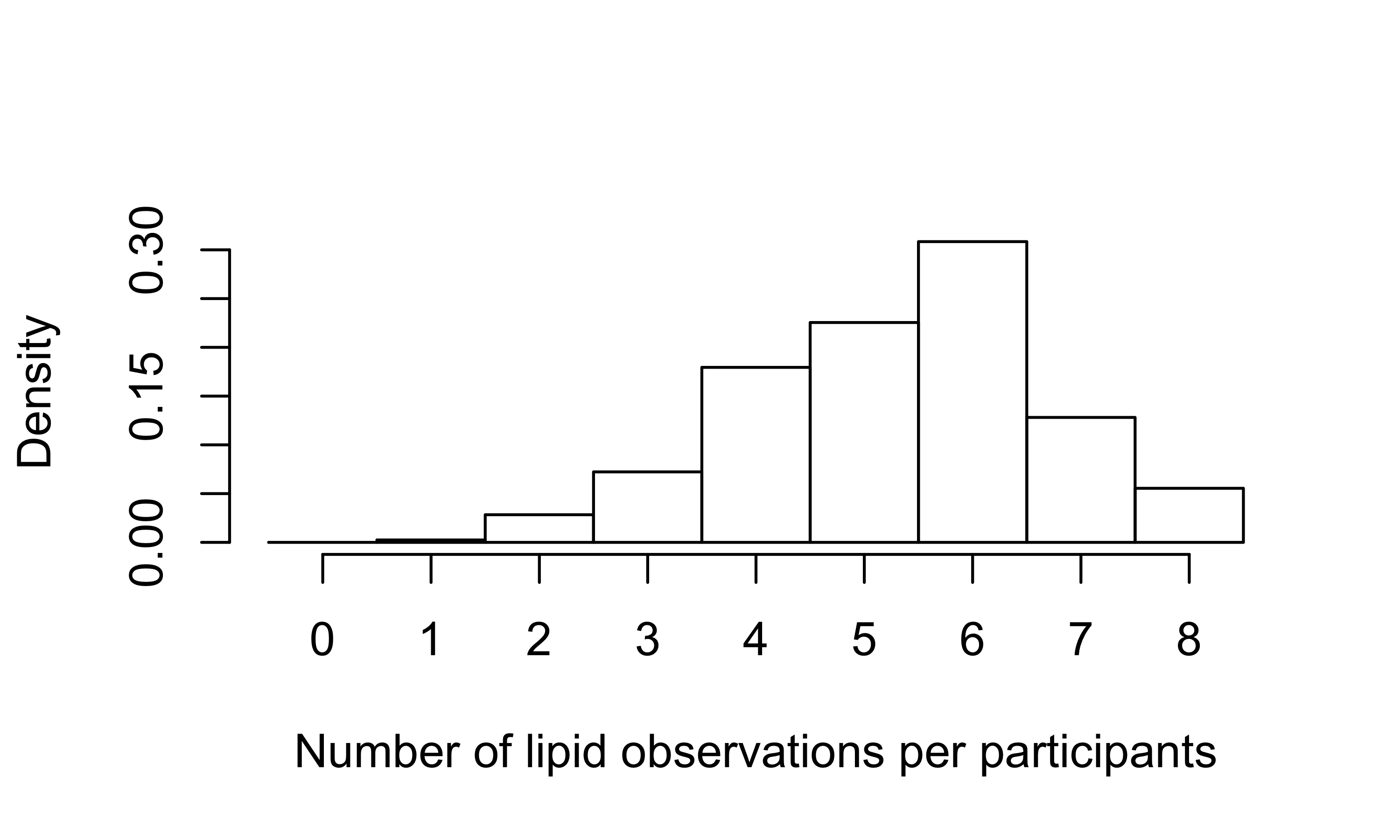


Average number of lipid observations per participants (sd): 5.31 (1.4)

Supplement: S2 File — For each lipid, green colour in histogram A denotes the lower GRS quartile (i.e. the 25th percentile); red colour, the upper GRS quartile (i.e. the 75th percentile); and white colour, the interquartile range. (i.e. the 50% of the data lying between upper and lower quartile). (DOCX) [file pone.0146081.s005.docx]
