# Supplementary material for: The Combined Effect of Common Genetic Risk Variants on Circulating Lipoproteins Is Evident in Childhood: A Longitudinal Analysis of the Cardiovascular Risk in Young Finns Study
Source: PLoS One. 2016 Jan 5;11(1):e0146081. doi: 10.1371/journal.pone.0146081 (PMC4701181; doi:10.1371/journal.pone.0146081)
Supplement: S1 File — a, b Limitations in the sampling size in 1989 are due to physical examinations and blood tests collected only in one centre (Turku). In 1992, the limitation was due to economic constraints. * Maximum of participants in the current study was 2442 (i.e. participants successfully genotyped in 2001). (DOCX) [file pone.0146081.s004.docx]

**Table S1**

Table A

| **Year** | **No.** | **Age** |  |  |  |  |  |  |  |  |  |  |  |  |  |  |
| --- | --- | --- | --- | --- | --- | --- | --- | --- | --- | --- | --- | --- | --- | --- | --- | --- |
| 1980 | 3596 | 3 | 6 | 9 | 12 | 15 | 18 |  |  |  |  |  |  |  |  |  |
| 1983 | 2991 |  | 6 | 9 | 12 | 15 | 18 | 21 |  |  |  |  |  |  |  |  |
| 1986 | 2799 |  |  | 9 | 12 | 15 | 18 | 21 | 24 |  |  |  |  |  |  |  |
| 1989^a^ | 632 |  |  |  | 12 | 15 | 18 | 21 | 24 | 27 |  |  |  |  |  |  |
| 1992^b^ | 891 |  |  |  |  | 15 | 18 | 21 | 24 | 27 | 30 |  |  |  |  |  |
| 2001 | 2620 |  |  |  |  |  |  |  | 24 | 27 | 30 | 33 | 36 | 39 |  |  |
| 2007 | 2159 |  |  |  |  |  |  |  |  |  | 30 | 33 | 36 | 39 | 42 | 45 |
| 2011 | 1999 |  |  |  |  |  |  |  |  |  |  | 34 | 37 | 40 | 46 | 49 |

Table B

|  |  | **Ages** | | | | | | | | | | | | | | | | | | | | | |
| --- | --- | --- | --- | --- | --- | --- | --- | --- | --- | --- | --- | --- | --- | --- | --- | --- | --- | --- | --- | --- | --- | --- | --- |
|  |  | **3** | **6** | **9** | **12** | **15** | **18** | **21** | **24** | **27** | **30** | **33** | **34** | **36** | **37** | **39** | **40** | **42** | **42** | **45** | **46** | **49** | **No. Total** |
| **Years** | **1980** | 363 | 397 | 420 | 425 | 426 | 383 |  |  |  |  |  |  |  |  |  |  |  |  |  |  |  | 2412 |
|  | **1983** |  | 337 | 364 | 376 | 375 | 327 | 263 |  |  |  |  |  |  |  |  |  |  |  |  |  |  | 2042 |
|  | **1986** |  |  | 339 | 340 | 340 | 298 | 268 | 246 |  |  |  |  |  |  |  |  |  |  |  |  |  | 1831 |
|  | **1989** |  |  |  | 56 | 51 | 47 | 46 | 44 | 41 |  |  |  |  |  |  |  |  |  |  |  |  | 285 |
|  | **1992** |  |  |  |  | 143 | 119 | 122 | 119 | 98 | 100 |  |  |  |  |  |  |  |  |  |  |  | 701 |
|  | **2001** |  |  |  |  |  |  |  | 299 | 336 | 359 | 372 |  | 362 |  | 334 |  |  |  |  |  |  | 2062 |
|  | **2007** |  |  |  |  |  |  |  |  |  | 296 | 314 |  | 324 |  | 354 |  | 349 |  | 302 |  |  | 1939 |
|  | **2011** |  |  |  |  |  |  |  |  |  |  |  | 234 |  | 262 |  | 286 |  | 301 |  | 291 | 289 | 1663 |
