# Supplementary material for: The Combined Effect of Common Genetic Risk Variants on Circulating Lipoproteins Is Evident in Childhood: A Longitudinal Analysis of the Cardiovascular Risk in Young Finns Study
Source: PLoS One. 2016 Jan 5;11(1):e0146081. doi: 10.1371/journal.pone.0146081 (PMC4701181; doi:10.1371/journal.pone.0146081)
Supplement: S2 Table — The gene name listed under ‘locus’ is either plausible biological candidate gene in the locus or the nearest annotate gene to the SNP. ‘chr’ denote chromosome. ‘Freq (%)’ denotes the risk allele frequency in the YF population, Adapted from Teslovitch et al. (2010)[23] and Tikkanen et al. (2011) [29]. (DOCX) [file pone.0146081.s007.docx]

**Table S3**

| **HDL-C (38 SNPs)** | **Triglycerides (25 SNPs)** | **LDL-C (14 SNPs)** |
| --- | --- | --- |
| **SNP beta chr/ locus Frq (%)** | **SNP beta chr/ locus Frq (%)** | **SNP beta Chr/locus Frq (%)** |
| rs4660293_A 0.48 1/PABPC4 22.1  rs2814944_G 0.49 6/C6orf106 21.7  rs4731702_A 0.59 7/KLF14 54.4  rs2923084_A 0.41 11/AMPD3 13.6  rs7134375_A 0.40 12/PDE3A 59.2  rs7134594_A 0.44 12/MVK 53.2  rs1532085_A 1.45 15/LIPC 42.4  rs3764261_A 3.39 16/CETP 73.6  rs2925979_C 0.45 16/CMIP 31.7  rs4148008_C 0.42 17/ABCA8 31.4  rs4129767_A 0.39 17/PGS1 36.5  rs737337_A 0.64 19/LOC55908 8.0  rs1800961_C 1.88 20/HNF4A 4.3  rs6065906_T 0.93 20/PLTP 14.6  rs1689800_A 0.47 1/ZNF648 26.9  rs4846914_A 0.61 1/GALNT2 46.1  rs12328675_C 0.68 2/COBLL1 9.8  rs2972146_G 0.46 2/IRS1 36.4  rs6450176_G 0.49 5/ARL15 14.6  rs605066_T 0.39 6/CITED2 47.9  rs1084651_A 1.95 6/LPA 17.4  rs9987289_G 1.21 8/PPP1R3B 15,5  rs2293889_G 0.44 8/TRPS1 70.9  rs581080_C 0.65 9/TTC39B 87  rs1883025_C 0.94 9/ABCA1 80.1  rs3136441_C 0.78 11/LRP4 22.7  rs4759375_T 0.86 12/SBNO1 9.2  rs4765127_T 0.44 12/ZNF664 28.4  rs838880_C 0.61 12/SCARB1 41.9  rs2652834_G 0.39 15/LACTB 76.3  rs16942887_A 1.27 16/LCAT 15.9  rs11869286_C 0.48 17/STARD3 32.1  rs7241918_T 1.31 18/LIPG 83.6  rs12967135_G 0.42 18/MC4R 82.4  rs7255436_A 0.45 19/ANGPTL4 51.5  rs386000_C 0.83 19/LILRA3 29.7  rs181362_C 0.46 22/UBE2L3 67  rs13107325_G 0.84 4/SLC39A8 98.7 | rs10195252_T 2.01 2/COBLL1 65.2  rs1042034_T 5.99 2/APOB 74.3  rs10761731_A 2.38 10/JMJD1C 63.1  rs11613352_C 2.70 12/LRP1 77.3  rs11649653_C 2.13 16/CTF1 61.3  rs11776767_C 2.01 8/PINX1 30.2  rs1260326_A 8.76 2/APOB 35.4  rs12678919_A 13.64 8/LPL 91.2  rs13238203_C 7.91 7/TYW1B 97.2  rs1495741_G 2.85 8/NAT2 75.1  rs17145738_C 9.32 7/MLXIPL 88.0  rs174546_A 3.82 11/FADS1-2-3 40.3  rs2068888_G 2.28 10/CYP26A1 54.1  rs2131925_T 4.94 1/ANGPTL3 72.6  rs2247056_C 2.99 6/HLA 76.2  rs2412710_A 7.00 15/CAPN3 3.1  rs2929282_T 5.13 15/FRMD5 6.2  rs2954029_A 5.64 8/TRIB1 54.3  rs439401_C 5.50 19/APOE 72.4  rs442177_T 2.25 4/KLH8 52.4  rs5756931_T 1.54 22/PL2G6 60.3  rs645040_T 2.22 3/MSL2L1 85.8  rs9686661_T 2.57 5/MAP3K1 14.3  rs964184_C 16.95 11/APOA1 14.1 | rs4299376_G 2.75 2/ABCG5/8 20.3  rs3757354_C 1.43 6/MYLIP 75.1  rs1800562_G 2.22 6/HFE 96.5  rs1564348_T 0.56 6/LPA 14.3  rs11220462_A 1.95 11/ST3GAL4 18.2  rs8017377_A 1.14 14/NYNRIN 37.2  rs6511720_G 6.99 19/LDLR 10.3  rs2479409_G 2.01 1/PCSK9 28.6  rs629301_T 5.65 1/SORT1 78.1  rs1367117_A 4.05 2/APOB 30.5  rs11136341_G 1.40 8/PLEC1 36.2  rs7206971_A 0.78 17/OSBPL7 48.1  rs4420638_G 7.14 19/APOE 25.1  rs6029526_A 1.39 20/TOP1 53.3 |
