# Supplementary material for: The Combined Effect of Common Genetic Risk Variants on Circulating Lipoproteins Is Evident in Childhood: A Longitudinal Analysis of the Cardiovascular Risk in Young Finns Study
Source: PLoS One. 2016 Jan 5;11(1):e0146081. doi: 10.1371/journal.pone.0146081 (PMC4701181; doi:10.1371/journal.pone.0146081)
Supplement: S3 Table — Childhood cutoffs apply to participants 19 years and younger. Chosen cutoffs are based on NCEP adult-panel treatment guidelines, NECP pediatric and adolescent guidelines and AAP and AHA pediatric guidelines (see methods). (DOCX) [file pone.0146081.s008.docx]

**S4 table**

|  | | **Normal Risk** | **High Risk** |
| --- | --- | --- | --- |
| **HDL-C** | **Childhoood** | > 0.91 | ≤ 0.91 |
|  | **Adulthood** | > 1.03 | ≤ 1.03 |
| **LDL-C** | **Childhoood** | < 4.14 | ≥ 4.14 |
|  | **Adulthood** | < 3.37 | ≤ 3.37 |
| **Triglycerides** | **Childhoood** | <1. 4 | ≤ 1.4 |
|  | **Adulthood** | < 1.7 | ≤ 1.7 |
