# Supplementary material for: The Combined Effect of Common Genetic Risk Variants on Circulating Lipoproteins Is Evident in Childhood: A Longitudinal Analysis of the Cardiovascular Risk in Young Finns Study
Source: PLoS One. 2016 Jan 5;11(1):e0146081. doi: 10.1371/journal.pone.0146081 (PMC4701181; doi:10.1371/journal.pone.0146081)
Supplement: S4 Table — Effect sizes β are in mmol/L for the number of risk allele differences between high and low wGRSs. Reported at each age are: Int (SE), the average lipid level in mmol/L (SE) in the high risk score group; β (SE), the difference in average lipid level in mmol/L for subjects in the low risk score group, the p-value for each cross-sectional association and the number of non-missing serum lipid observations considered in each regression (Nobs). (*: For each sex, the number of risk alleles difference between high and low wGRS groups are calculated as: median (Number of alleles in the High wGRS)–median ((Number of alleles in the Low wGRS)). (DOCX) [file pone.0146081.s009.docx]

**S5 Table**

|  | | **wGRS** | **Number of**  **risk alleles** | **Average Lipid Levels** | | | |
| --- | --- | --- | --- | --- | --- | --- | --- |
|  | |  |  | **3 years** | **15 years** | **24 years** | **45/46 years** |
| **LDL-C** | Males | High  Low | 16.72  12.19 | Int: 3.57 (0.13)  β: -0.49 (0.12)  P-val=0.005  Nobs=173 | Int: 3.01 (0.11)  β: -0.42(0.12)  P-val=9.1*10^-7^  Nobs=617 | Int: 3.23 (0.13)  β: -0.45 (0.11)  P-val=8.3*10^-6^  Nobs=313 | Int: 3.68(0.14)  β: -0.56 (0.10)  P-val=9.7*10^-8^  Nobs=421 |
|  | Females | High  Low | 16.68   12.55 | Int: 3.78 (0.11)  β:-0.35 (0.11)  p-val=0.023  Nobs=184 | Int: 3.31 (0.10)  β: -0.39 (0.09)  P-val=3.8*10^-6^  Nobs=711 | Int: 3.16 (0.10)  β: -0.41 (0.11)  P-val=2.1*10^-5^  Nobs=397 | Int: 3.57 (0.11)  β: -0.51 (0.10)  P-val=2.0*10^-8^  Nobs=475 |
| **HDL-C** | Males | High  Low | 48.08  40.99 | Int: 1.56 (0.08)  β: -0.15 (0.04)  P-val=0.025  Nobs=161 | Int: 1.48 (0.06)  β: -0.15 (0.05)  P-val=5.8*10^-6^  Nobs=594 | Int: 1.3 (0.08)  β: -0.13 (0.03)  P-val=0.01  Nobs=293 | Int: 1.40 (0.07)  β: -0.16 (0.07)  P-val=1.3*10^-4^  Nobs=415 |
|  | Females | High  Low | 48.00  41.01 | Int: 1.56 (0.06)  β: -0.16 (0.05)  P-val=0.004  Nobs=174 | Int: 1.62 (0.06)  β: -0.15 (0.04)  P-val=2.5*10^-6^  Nobs=688 | Int: 1.56 (0.04)  β: -0.13 (0.04)  P-val=0.01^7^  Nobs=373 | Int: 3.01 (0.08)  β: -0.17 (0.05)  P-val=3.7*10^-5^  Nobs=446 |
| **Triglycerides** | Males | High  Low | 28.99  23.02 | Int: 0.70 (0.08)  β: -0.17 (0.05)  P-val=0.001  Nobs=173 | Int: 0.96 (0.1)  β: -0.23 (0.03)  P-val=4.3*10^-8^  Nobs=620 | Int: 1.5 (0.1)  β: -0.46 (0.03)  P-val=5.8*10^-5^  Nobs=315 | Int: 1.77 (0.15)  β: -0.53 (0.04)  P-val=3.5*10^-6^  Nobs=439 |
|  | Females | High  Low | 28.99  23.07 | Int: 0.75 (0.09)  β: -0.19 (0.07)  P-val=8.5*10^-6^  Nobs=186 | Int: 0.93(0.08)  β: -0.17(0.05)  P-val=1.1*10^-6^  Nobs=717 | Int: 1.33 (0.07)  β: -0.20 (0.06)  P-val=0.0001  Nobs=393 | Int: 1.65 (0.08)  β: -0.23 (0.07)  P-val=1.5*10^-8^  Nobs=470 |
