# Supplementary material for: The Combined Effect of Common Genetic Risk Variants on Circulating Lipoproteins Is Evident in Childhood: A Longitudinal Analysis of the Cardiovascular Risk in Young Finns Study
Source: PLoS One. 2016 Jan 5;11(1):e0146081. doi: 10.1371/journal.pone.0146081 (PMC4701181; doi:10.1371/journal.pone.0146081)
Supplement: S1 Table — Abbreviations: HDL-C, high-density lipoprotein cholesterol; LDL-C, low-density lipoprotein cholesterol, wGRS-HDL; HDLwGRS3 continuous combined genetic risk score for HDL (38 SNPs); wGRS-HDL, LDLwGRS14, continuous combined genetic risk score for LDL-C (14 SNPs); wGRS-TG, TGwGRS24, continuous combined genetic risk score for triglycerides (24 SNPs); adult.age, age of participants at latest adult follow up (2001, 2007 or 2011),Δ BMI, change in BMI z-score between childhood and adulthood as described in Methods section. * For HDL-C and LDL-C, the effect of wGRSs is in mmol/L per 1-sd change in wGRS, and the effect of Δ BMI is in mmol/L per 1-sd change in Δ BMI. For triglycerides, the effect of wGRSs is in odds ratio per 1-sd change in wGRS, and the effect of Δ BMI is in odds ratios per 1-sd change in Δ BMI. (PDF) [file pone.0146081.s006.pdf]

**Adult LDL-C model** (N=2100)	**AIC/BIC**	**β (se)*, p-val**
**Model 1 :**	3686/3716	$\beta_{\text{wGRS-LDL:}}$ 0.029 (0.02),p<$2\text{x}10^{-16}$ *
Adult.LDL~ sex+ adult.age+ wGRS-LDL+Δ BMI		$\beta\ \Delta_{\text{BMI :}}$ 0.09 (0.01), p=$1.3\text{x}10^{-6}$ *
+ wGRS-LDL*Δ BMI		$\beta_{\text{wGRS-LDL*}\Delta}\Delta_{\text{BMI:}}$-0.02(0.01), p=0.15
**Model 2:**	3685/3723	$\beta_{\text{wGRS-LDL:}}$ 0.20 (0.02), p<$1\text{x}10^{-16}$ *
Adult.LDL~ sex+ adult.age+wGRS-LDL+Δ BMI		$\beta\ \Delta_{\text{BMI :}}$ 0.09(0.02), p<$1.0\text{x}10^{-7}$*
**Likelihood Ratio Test : Model 1 vs model 2**		
χ2(df=1)=2.04 p=0.15		

**Adult HDL-C model** (N=2034)	**AIC/BIC**	**β (se)*, p-val**
**Model 3 :**	486/418	$\beta_{\text{wGRS-HDL:}}$ 0.06(0.007), p <$2\text{x}10^{-16}$ *
Adult.HDL ~ sex+adult.age+wGRS-HDL+Δ BMI		$\beta\ \Delta_{\text{BMI :}}$ -0.09 (0.007), p= 0.046 *
+ wGRS-HDL*Δ BMI		$\beta_{\text{wGRS-HDL*}}\Delta_{\text{BMI:-}}$0.003 (0.006),p=0.62
**Model 4:**	488/525	$\beta_{\text{wGRS-HDL:}}$ 0.06 (0.007), p<$1\text{x}10^{-16}$
Adult.HDL ~sex+adult.age +wGRS-HDL+Δ BMI		$\beta\ \Delta_{\text{BMI :}}$ - 0.09 (0.07), p<$2\text{x}10^{-16}$
**Likelihood Ratio Test: Model 3 vs model 4**		
χ2(df=1)=0.24 p=0.62		

**Adult triglycerides model** (N=2062)	**AIC/BIC**	**β (se)*, p-val**
**Model 5 :**	2645/2876	$\beta_{\text{wGRS-TG:}}$ 1.029 (0.02),p<$2\text{x}10^{-8}$ *
Log(Adult.TG)~sex+adult.age+wGRS-TG+ΔBMI		$\beta\ \Delta_{\text{BMI :}}$ 1.09 (0.01), p=$2.1\text{x}10^{-5}$ *
wGRS- TG*Δ BMI		$\beta_{\text{wGRS-TG*}\Delta}\Delta_{\text{BMI:-}}$1.002(0.01), p=0.3
**Model 6:**	3685/3723	$\beta_{\text{wGRS-TG:}}$ 1.029 (0.02), p<$1\text{x}10^{-8}$ *
Log(Adult.TG)~sex+adult.age+wGRS-TG+Δ BMI		$\beta\ \Delta_{\text{BMI :}}$ 1.08 (0.01), p<$1.0\text{x}10^{-9}$*
**Likelihood Ratio Test : Model 5 vs model 6**		
χ2(df=1)=1.17 p=0.11		
